# Exploring alterations in hematological and biochemical parameters, enzyme activities and serum cortisol in *Besnoitia besnoiti* naturally infected dairy cattle

**DOI:** 10.1186/s13071-021-04626-4

**Published:** 2021-03-15

**Authors:** Luca Villa, Alessia Libera Gazzonis, Sergio Aurelio Zanzani, Silvia Mazzola, Alessia Giordano, Maria Teresa Manfredi

**Affiliations:** Department of Veterinary Medicine, Università degli Studi di Milano, Via dell’Università 6, 26900 Lodi, Italy

**Keywords:** Bovine besnoitiosis, Dairy cattle, Hematology, Biochemistry, Enzyme activities, Cortisol

## Abstract

**Background:**

*Besnoitia besnoiti* is an Apicomplexan protozoa causative of bovine besnoitiosis, a chronic and debilitating disease of cattle, with a variety of pathological findings that could alter some laboratory parameters. A study was conducted in a bovine besnoitiosis endemically infected dairy herd located in Italy characterized by high intra-herd seroprevalence and cattle with clinical signs of the disease. In the study, alterations in laboratory parameters, i.e. hematological and biochemical parameters, enzyme activities and serum cortisol levels, in *Besnoitia besnoiti* naturally infected cows were investigated in depth.

**Methods:**

Laboratory parameters in 107 cows, of which 61 were seronegative and 46 were seropositive to *B. besnoiti*, including 27 with clinical signs of bovine besnoitiosis, were compared. Generalized linear models were used to evaluate the effect of *Besnoitia* infection on the considered laboratory parameters.

**Results:**

Hematological analyses revealed that *B. besnoiti* infection determined a significant alteration to the leukocyte differential, with a higher percentage of granulocytes and a lower percentage of lymphocytes in seropositive and clinically affected animals (Mann–Whitney U-test, *P* = 0.022); erythrocyte and platelet counts did not show any difference between the considered groups of cows. Biochemistry tests evidenced that the parasite infection influenced serum protein values in seropositive cows and glutamate dehydrogenase values in clinically affected animals. No or only slight differences were revealed for all of the other biochemical and enzyme activity parameters in *B. besnoiti*-infected animals. In addition, despite the lack of statistical significance, seropositive and clinically affected cows evidenced higher concentrations of serum cortisol values compared to seronegative animals.

**Conclusions:**

Although physiological, pathological and farm-related factors could have influenced the results in investigated animals, further studies involving more animals from different farms would be advisable to infer the role of *B. besnoiti* on these alterations, since laboratory parameters could help veterinarians in the diagnosis of bovine besnoitiosis in cattle.
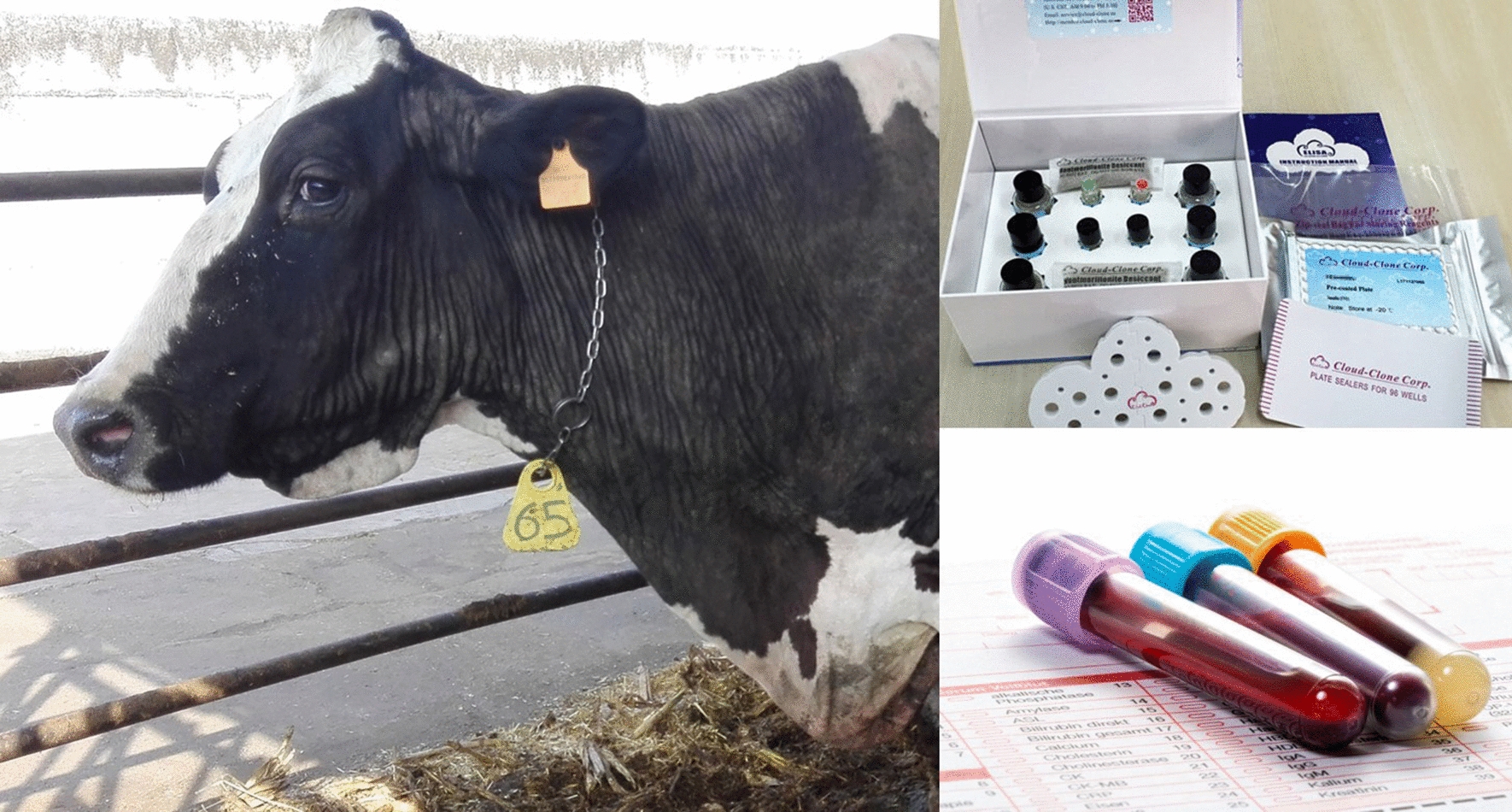

## Background

Bovine besnoitiosis is a parasitic disease caused by the cystogenic coccidian parasite *Besnoitia besnoiti*. In Europe, it is considered an emerging or re-emerging disease, with an increasing geographical distribution and the number of cases of infection [1]. In Italy, in the last decade, outbreaks of bovine besnoitiosis were reported, particularly in the northern regions [2–5].

Bovine besnoitiosis is a chronic and debilitating disease characterized by both cutaneous and systemic manifestations, compromising animal welfare and responsible for economic losses on affected farms [1, 6, 7]. The disease progresses in two sequential clinical phases. Initially, the intense multiplication of tachyzoites within endothelial cells causes vasculitis, hyperplasia, thrombosis, necrosis of venules and arterioles, and increased vascular permeability, subsequently resulting in congestion, hemorrhages and infarcts. The acute phase is thus characterized by hyperthermia, edemas and other non-specific clinical signs, including depression, swelling of the superficial lymph nodes, arrest of rumination, weight loss, anorexia, photophobia, epiphora, ocular and nasal discharge, tachycardia and tachypnea. The subsequent chronic phase is due to the slow replication of bradyzoites inside tissue cysts with tropism for connective tissues, surrounded by granulomatous inflammation. The skin becomes progressively thickened and wrinkled with alopecia, seborrhea, and hyperkeratosis, with the presence of pathognomonic subcutaneous thick-walled tissue cysts. Chronically infected cattle show loss of weight and progressive deterioration of the body condition [6, 8]. The pathological findings occurring during both the acute and chronic phases of bovine besnoitiosis can lead to alterations in laboratory parameters.

A few studies focused on the hematological and biochemical changes during *B. besnoiti* infection. One study investigated both the impact of naturally acquired acute, subacute and chronic besnoitiosis on these parameters in three Simmental heifers and two Limousin cows and compared the changes in seronegative and seropositive Limousins from a German cow–calf operation, analyzing a high number of samples from a limited number of animals (224 samples from 9 Simmental and 75 samples from 54 Limousin cattle) [9]. Alterations in blood parameters of 11 beef cattle from an outbreak of bovine besnoitiosis in southwestern Spain [10] and in a naturally infected bull from South Africa were also reported [11]. Most of the studies concerned beef cattle, except that of Alshehabat et al. [12] in which selected metabolic biochemical parameters including enzyme activities associated with *B. besnoiti* infection were evaluated in dairy cattle from Jordan. Moreover, hematology and biochemistry changes were observed in goats affected by naturally acquired caprine besnoitiosis in Iran [13, 14].

To the best of our knowledge, no studies to date investigated the cortisol concentration in cows affected with bovine besnoitiosis. Cortisol is a cholesterol-derived steroid synthesized in the adrenal cortex under the control of the HPA (hypothalamic–pituitary–adrenal) axis that reacts to a wide range of stimuli from both internal and external origin. This glucocorticoid is the primary hormone involved in the stress response: its main action is to activate biological functions to respond to stress and to restore homeostasis after exposure to stressors. It is commonly used as an indicator of stress and welfare in animals [15]. Prolonged elevation of cortisol influences negatively the immune response, causing suppression of the immune system and increased susceptibility to disease; it also has adverse effects on the reproductive success of animals [16]. It was also reported that during immunosuppression *B. besnoiti* proliferation in cattle might be facilitated [17].

Considering that the spread of bovine besnoitiosis could be underestimated [1, 18] and that no studies to date focused on alterations in laboratory parameters in Holstein Friesian dairy cows under intensive farming conditions, the main goal of this study was to investigate alterations in hematological and biochemical parameters, including muscle and liver enzyme activity, and variations in serum cortisol levels in *B. besnoiti* naturally infected cows from an intensive dairy herd endemically affected by bovine besnoitiosis. Further aims were (i) to evaluate if the infection could be predicted by variations in the laboratory parameters investigated and (ii) to verify the effects of the parasite infection on health status and welfare of infected animals.

## Methods

### Background

The study was conducted in a bovine besnoitiosis endemically infected dairy herd hosting 217 Holstein Friesian cattle located in the province of Brescia (Lombardy, northern Italy). A serological screening for *B. besnoiti* was performed on the whole herd; Western Blot-positive results were considered as *B. besnoiti* seropositive animals [5]. In addition, a number of the seropositive animals were clinically examined to reveal symptoms and lesions of besnoitiosis. The herd resulted affected by high intra-herd *B. besnoiti* seroprevalence (23.5% increasing up to 43.5% considering only cows) and a number (*n* = 27) of the seropositive animals suffered from clinical signs of the chronic phase of the disease; indeed, clinically affected cattle evidenced the presence of tissue cysts in at least one of the typical localizations (sclera, vulva, or skin). Moreover, a case of severe systemic besnoitiosis in a chronically affected cow was also recorded [5]. Besides, a form of generalized demodectic mange in two cows co-infected with *B. besnoiti* was also reported [19].

### Sample and data collection

Overall, 107 cows from the infected herd, 46 seropositive to *B. besnoiti* and 61 seronegative, were included in the study. During the sampling for the serological screening [5], blood samples were also collected in tubes with EDTA for hematological analyses. In addition to the aliquot of the sera collected for serological analyses, a second aliquot was stored at − 20 °C for biochemistry, including liver and muscle enzyme activity analyses, and a third aliquot was stored at − 20 °C for cortisol determination. Epidemiological data, including individual data (breed, sex, productive category, age and origin) and reproductive parameters (number of parturitions and phase of lactation) were noted.

### Hematology

Hematological analyses were performed on blood samples preserved in tubes with EDTA within 24 h from the collection time, using the Cell-Dyn 3500 analyzer (Abbott Laboratories, Abbott Park, IL, USA). The following hematological parameters were assessed: red blood cells (RBC), hemoglobin (Hb), hematocrit (Ht), mean corpuscular volume (MCV), mean corpuscular hemoglobin (MCH), mean corpuscular hemoglobin concentration (MCHC), red cell distribution width (RDW), white blood cells (WBC), lymphocytes, granulocytes, platelet count (PLT), mean platelet volume (MPV), plateletcrit (Pct) and platelet distribution width (PDW).

### Biochemistry and enzyme activity

Biochemistry and enzyme activity analyses were carried out on serum using the automated analyzer BT3500 (Biotecnica Instruments S.p.a., Rome, Italy) using reagents, controls and calibrators provided by Futurlab Srl (Limena, Italy) and Randox Laboratories Ltd. (Crumlin Co., Antrium, UK; exclusively for BOHB and NEFA). The following analytes were measured (acronyms [as needed] followed by methods in parenthesis): albumin (bromochresol green), aspartate aminotransferase (AST, kinetic IFCC), β-hydroxybutyrate (BOHB, d-3-hydroxybutyrate dehydrogenase), calcium (Ca, orthocresoftaleine), magnesium (Mg, xylidyl blue), creatine kinase (CK, kinetic IFCC), glutamate dehydrogenase (GLDH, kinetic IFCC), lactate (LO-POD), lactate dehydrogenase (LDH, kinetic IFCC), non-esterified fatty acid (NEFA, ACS-ACOD), phosphorous (P, phosphomolibdate), total bilirubin (modified Jendrassik–Grof assay), total proteins (modified biuret). Globulin concentration was calculated by subtracting albumin concentration from total proteins, whereas the albumin:globulin ratio (A/G) was calculated by dividing albumin concentration by globulin concentration.

### Cortisol determination

Cortisol concentration was assessed in serum samples using a commercially available enzyme-linked immunosorbent assay kit (Cortisol ELISA kit; article. CEA462Ge; Cloud Clone Corp., Katy, TX, USA). Sera stored at − 20 °C were analyzed after few days but always within 1 month of storage to avoid loss of bioactivity and contamination, according to the manufacturer’s instructions. Each sample was prepared in duplicate, and absorbance was measured at a wavelength of 405 nm, using a LabSystems Multiskan Ex microplate reader (Nepean, ON, Canada), according to the relevant standard curves. The mean recovery was 102.8% ± 10.8, The average intra- and inter-assay coefficients of variation were 5.7 and 7.6%, respectively; the assay sensitivity was 5.15 ng/ml. The laboratory technician was blinded to the hypotheses and conditions.

### Statistical analysis

Values of hematology, biochemistry, enzyme activities and serum cortisol of seronegative and *B. besnoiti* seropositive dairy cattle were compared statistically. The same laboratory parameters were also compared between the groups of seronegative or asymptomatic seropositive cows and animals with clinical signs of chronic besnoitiosis. To assess normality, the Shapiro–Wilk test was used. To compare the two groups, the non-parametric Mann–Whitney U-test was performed. The level of significance was set at *P* values of < 0.05.

Separate generalized linear models (GLMs) with linear distribution were performed for each analyte to verify the influence of *B. besnoiti* infection on hematological and biochemical parameters, enzyme activities and serum cortisol levels (dependent variables). As predictor, the outcome of *B. besnoiti* infection was considered both as (i) seropositivity for *B. besnoiti* (dichotomous variable: presence* vs* absence of antibodies based on Western Blot results) and (ii) clinical signs of bovine besnoitiosis (dichotomous variable: seronegative or asymptomatic seropositive animals* vs* clinically affected cows); these outcomes were evaluated individually, entered as independent variables in separate models. For the models containing the variable “seropositivity for *B. besnoiti*,” all examined animals were included. For the models containing the variable “clinical signs of bovine besnoitiosis,” 14 seropositive animals were excluded from the analysis, since it was not possible to perform a clinical examination and thus ascertain their clinical status. In addition, to consider the influence of the physiological status of the cows on the considered laboratory parameters, number of parturitions (dichotomous variable: primiparous* vs* multiparous cows) and phase of lactation (ordinal variable: early, mid, late and dry) were also entered in each model as independent variables. In addition, the two-way interactions between *B. besnoiti* infection (seropositivity for *B. besnoiti* or clinical signs of bovine besnoitiosis), number of parturitions and phase of lactation were considered, to verify the overall influence of both the parasite infection and the physiological status of the cows on the considered laboratory parameters. All variables and their two-way interactions were entered in multivariate models developed through a backward selection procedure (significance level to remove variables from the model = 0.05), based on Akaike’s information criterion (AIC) values. When retained in the final models, the estimated means of the variable “phase of lactation” and the interactions were compared through pairwise comparisons. Statistical analysis was performed using IBM SPSS Statistics for Windows version 25.0 software (IBM Corp., Armonk, NY, USA).

## Results

In this study, laboratory parameters of 107 cows, 61 of which were seronegative and 46 seropositive for *B. besnoiti*, including 27 with clinical signs of chronic bovine besnoitiosis, were investigated.

All sampled animals were female Holstein Friesian cows and all were born in the farm. The mean age of sampled cows was 46.8 months (standard deviation [SD] 17.7, minimum–maximum [Min–Max] 25.4–115.6 months). Forty-six of the cows were primiparous, with the remaining having had between two and six parturitions. Ninety-five animals were lactating at the time of sampling: 31 of them were in the early, 37 in the mid and 27 in the late phase of lactation, respectively; the remaining 12 animals were in the dry phase.

The results of hematological and biochemical parameters, enzyme activities and serum cortisol levels are summarized in Table 1. Descriptive statistics were assessed according to the groups of seronegative and seropositive cows based on the Western Blot results for *B. besnoiti*; in addition, among seropositive animals, a subgroup of cows presenting clinical signs of bovine besnoitiosis was considered. The laboratory parameters results were also assessed according to the number of parturitions (primiparous and multiparous cows) and the phase of lactation (early, mid, late and dry), as reported in Additional file 1: Table S1 and Additional file 2: Table S2, respectively. For all of the considered laboratory parameters, specific reference ranges for dairy cows were used.

**Table 1:** Descriptive statistics of hematological, biochemical, enzyme activity analyses and cortisol determination, according to the serological and clinical status of cows in a dairy cattle herd endemically infected by bovine besnoitiosis

Parameter	Unit	Seronegative^a^		Seropositive^a^				Range
		Mean (SD)	Min–Max	Overall		Clinically affected^a^		
				Mean (SD)	Min–Max	Mean (SD)	Min–Max	
Hematology								
RBC	×10^6^/µl	6.11 (0.73)	4.67–8.53	6.15 (0.67)	4.80–7.14	6.07 (0.66)	4.80–7.02	6–10
Hb	g/dl	12.46 (1.55)	8.86–15.88	12.18 (1.75)	3.81–14.89	12.04 (2.01)	3.81–14.45	8–12
Ht	%	26.77 (2.60)	20.70–31.80	27.35 (2.73)	22.20–32.40	26.69 (2.30)	22.20–30.80	20–35
MCV	µ^3^	44.08 (3.97)	32.00–56.00	44.70 (3.22)	38.00–50.00	44.30 (3.45)	39.00–50.00	40–50
MCH	pg	20.57 (2.91)	14.74–27.48	19.92 (3.12)	7.62–24.96	19.92 (3.46)	7.62–24.96	15–20
MCHC	g/dl	46.74 (5.62)	35.95–62.35	44.64 (6.34)	17.17–56.71	45.08 (7.17)	17.17–56.71	30–40
RDW	%	15.23 (0.99)	13.40–17.50	15.22 (0.79)	13.80–16.90	15.20 (0.84)	13.90–16.90	15–17
WBC	×10^3^/µl	9.35 (6.31)	2.70–48.10	9.69 (6.58)	4.70–37.20	9.70 (6.33)	4.70–35.80	6–12
Lymphocytes	%	53.52 (13.69)	11.00–97.00	*45.78* (*18.53*)^b^	12.00–88.00	46.48 (18.46)	12.00–86.00	55–85
Granulocytes	%	46.48 (13.69)	3.00–89.00	*54.22* (*18.53*)^b^	12.00–88.00	53.52 (18.46)	14.00–88.00	15–45
PLT	×10^3^/µl	307.77 (115.04)	17.10–490.00	286.41 (106.87)	90.00–557.00	286.74 (111.46)	90.00–557.00	200–800
MPV	µ^3^	13.43 (35.35)	8.20–285.00	8.82 (0.32)	8.10–9.50	8.80 (0.30)	8.30–9.40	5–9
Pct	%	0.28 (0.09)	0.07–0.43	0.25 (0.09)	0.08–0.47	0.25 (0.10)	0.08–0.47	0.1–0.4
PDW	%	13.39 (13.00)	9.70–113.00	11.66 (1.05)	9.70–14.20	11.63 (1.17)	9.70–14.20	10–14
Biochemistry								
Total proteins	g/dl	8.73 (1.11)	6.30–11.50	8.34 (1.04)	5.60–10.50	8.28 (1.06)	5.60–10.50	5.9–7.7
Albumin	g/dl	2.96 (0.52)	2.00–4.20	3.12 (0.60)	2.00–4.70	2.93 (0.52)	2.00–3.90	2.7–4.3
Globulin	g/dl	5.78 (1.34)	2.90–8.90	5.22 (1.10)	3.20–7.70	5.36 (0.92)	3.60–6.80	1.6–5
A/G ratio		0.56 (0.22)	0.26–1.28	0.63 (0.21)	0.31–1.19	0.56 (0.13)	0.37–0.82	0.7–1.5
Total bilirubin	mg/dl	0.56 (0.36)	0.09–1.63	0.62 (0.84)	0.13–5.26	0.72 (1.05)	0.13–5.26	0.1–0.3
Ca	mg/dl	9.56 (0.74)	7.00–11.10	9.54 (0.72)	7.10–10.80	9.40 (0.81)	7.10–10.80	7.9–10
P	mg/dl	6.43 (1.21)	3.90–9.70	6.18 (1.21)	4.00–10.40	6.13 (1.35)	4.00–10.40	4.6–9
Mg	mg/dl	2.14 (0.35)	1.38–3.10	2.15 (0.35)	1.22–2.93	2.17 (0.36)	1.22–2.65	1.4–2.3
NEFA	mmol/l	0.32 (0.24)	0.06–1.05	0.34 (0.20)	0.09–0.76	0.33 (0.21)	0.12–0.76	0.4–0.8
BOHB	mmol/l	0.64 (0.24)	0.29–1.54	0.60 (0.28)	0.05–1.48	0.65 (0.31)	0.05–1.48	0.03–0.3
Lactate	mmol/l	2.82 (0.92)	1.47–5.23	2.81 (0.94)	1.10–5.39	2.78 (1.02)	1.10–5.39	< 2
Enzymes								
AST	U/l	74.40 (33.17)	43.00–252.00	74.07 (21.24)	47.00–159.00	75.26 (23.16)	48.00–159.00	48–100
CK	U/l	303.58 (1278.29)	28.00–9971.00	173.76 (332.28)	37.00–2182.00	145.59 (161.06)	39.00–782.00	44–228
LDH	U/l	870.42 (261.21)	434.00–2173.00	866.44 (253.36)	464.00–2113.00	904.44 (289.32)	535.00–2113.00	500–1500
GLDH	U/l	33.42 (51.46)	6.40–367.00	31.49 (31.09)	0.00–137.70	36.69 (36.10)	0.00–137.70	< 20
Cortisol	ng/ml	6.81 (6.41)	0.77–35.48	9.11 (9.51)	0.56–43.86	8.54 (11.36)	0.56–43.86	< 10

All hematology parameters are defined in Section Hematology; all biochemistry and enzymes parameters are defined in Section Biochemistry and enzyme activity of the Methods

Min–Max, Minimum–Maximum values; SD, standard deviation

^a^Serological status (seronegative or seropositive) was determined according to Western Blot results. Clinically affected cows refer to those animals showing clinical signs of the disease

^b^Laboratory parameters significantly (*P* < 0.05) different vs. seronegative cows (Mann–Whitney *U* Test) are highlighted in italics

### Hematology

Mean values for RBC, Hb, Ht and the erythrocyte indices (MCV, MCH, MCHC and RDW) were similar between seronegative, seropositive and clinically affected cows (Table 1). However, considering overall both seronegative and* B. besnoiti* seropositive animals, alterations in some of these parameters were revealed. In particular, mean RBC (6.13 ± 0.70 ×10^6^/µl), Ht (27.02 ± 2.66%) and RDW (15.23 ± 0.90%) values were low and mean MCV (44.35 ± 3.66 µ^3^) and MCH (20.29 ± 3.01 pg) values were high, but remained within the limits of the reference interval, respectively (Table 1).

WBC counts were also similar in the considered groups of animals (seronegative, seropositive and clinically affected cows), and mean values fell within the reference interval (Table 1). Nonetheless, the leukocyte differential was altered, with a higher percentage of granulocytes and a lower percentage value of lymphocytes in seropositive (54.22 ± 18.53 and 45.78 ± 18.53%, respectively) and clinically affected (53.52 ± 18.46 and 46.48 ± 18.46%, respectively) cows, particularly in primiparous seropositive cows (62.19 ± 16.51 and 37.81 ± 16.51%, respectively) and primiparous animals with clinical signs of besnoitiosis (56.77 ± 23.61 and 43.23 ± 23.61%, respectively), in comparison to seronegative and multiparous ones (Additional file 1: Table S1). Indeed, while the values for seronegative animals were equally distributed in terms of the reference interval for the leukocyte differential, 69.6% of seropositive cows, increasing up to 87.5% if considering only primiparous cows, showed a mean concentration of lymphocytes and granulocytes below and above the reference range, respectively.

The mean PLT, MPV, Pct and PDW values were slightly lower in seropositive (286.41 ± 106.87 ×10^3^/µl, 8.82 ± 0.32 µ^3^, 0.25 ± 0.09% and 11.66 ± 1.05%, respectively) and clinically affected cows (286.74 ± 111.46 ×10^3^/µl, 8.80 ± 0.30 µ^3^, 0.25 ± 0.10% and 11.63 ± 1.17%, respectively) than in seronegative animals (307.77 ± 115.04 ×10^3^/µl, 13.43 ± 35.35 µ^3^, 0.28 ± 0.09%, and 13.39 ± 13.00%, respectively), but mean values for all groups of cows fell within the reference intervals (Table 1).

### Biochemistry and enzyme activity

Regarding serum total protein concentration, the mean of total proteins (8.57 ± 1.09 g/dl) was elevated in all cows; albumin (3.03 ± 0.55 g/dl) and globulin (5.54 ± 1.27 g/dl) concentrations were at the lower and upper limit of the range, respectively, with a slightly lower value of the A/G ratio (0.59 ± 0.22). In addition, seropositive (8.34 ± 1.04 g/dl) and clinically affected (8.28 ± 1.06 g/dl) cows showed lower values of total protein than seronegative animals (8.73 ± 1.11 g/dl); globulin concentrations were lower in seropositive (5.22 ± 1.10 g/dl) and clinically affected (5.36 ± 0.92 g/dl) animals than seronegative cows (5.78 ± 1.34 g/dl). Albumin (3.12 ± 0.60 g/dl) and A/G ratio (0.63 ± 0.21) values were higher in seropositive cows, but slightly lower in clinically affected animals (2.93 ± 0.52 g/dl and 0.56 ± 0.13), in comparison to seronegative ones (2.96 ± 0.52 g/dl and 0.56 ± 0.22, respectively). Values of serum protein differed according to the lactation phase: in particular, total protein values (9.13 ± 1.02 g/dl) were highest in the late phase, albumin (3.68 ± 0.34 g/dl) and A/G ratio (0.87 ± 0.26) were highest in dry cows, whereas globulin values were highest in the late phase of lactation (6.14 ± 1.27 g/dl) but lowest in dry animals (4.59 ± 1.35 g/dl).

Total bilirubin was elevated over the range limit in all cows (0.58 ± 0.61 mg/dl), with higher values in seropositive (0.62 ± 0.84 mg/dl) and clinically affected animals (0.72 ±  1.05 mg/dl) than in seronegative ones (0.56 ± 0.36 mg/dl).

In terms of minerals, the mean concentration of Ca, P, and Mg were within the limits, with similar values in seropositive and seronegative cows (Table 1).

Mean NEFA, BOHB and lactate values were similar between the groups of cows according to *B. besnoiti* seropositivity and clinical signs (Table 1). However, considering mean concentrations in all animals, values of BOHB (0.62 ± 0.25 mmol/l) and lactate (2.82 ± 0.93 mmol/l) were above the reference range, while NEFA values (0.33 ± 0.22 mmol/l) fell within the reference interval.

Regarding enzyme activities, AST and LDH values fell within the normal range and showed similar values in all groups of animals (Table 1), with only a slightly higher value in clinically affected animals (75.26 ± 23.16 and 904.44 ± 289.32 U/l, respectively) compared with seronegative cows (74.40 ± 33.17 and 870.42 ± 261.21 U/l), respectively. CK level was elevated over the upper limit in seronegative animals (303.58 ± 1278.29 U/l), while the mean value was within the reference interval in seropositive cows (173.76 ± 332.28 U/l). GLDH values were above the reference limit in all the three groups of cows (32.58 ± 43.65 U/l), with a slight higher value in clinically affected animals (36.69 ± 36.10 U/l).

### Cortisol determination

Seropositive (9.11 ± 9.51 ng/ml) and clinically affected (8.54 ± 11.36 ng/ml) cows evidenced higher values of cortisol when compared to seronegative animals (6.81 ±  6.41 ng/ml). Cortisol levels were generally higher in the early lactation phase (9.13 ± 12.37 ng/ml) and in dry cows (10.96 ± 6.27 ng/ml), compared to cows in the mid (5.62 ± 4.87 ng/ml) and late (8.27 ± 5.76 ng/ml) phase of lactation. In contrast, mean values were similar in primiparous (7.15 ± 7.33 ng/ml) and multiparous (8.04 ± 8.52 ng/ml) cows. However, overall mean cortisol levels (7.92 ± 8.09 ng/ml) were generally elevated in all animals with respect to the recommended reference value (5–10 ng/ml).

### Statistical analysis

Values of hematology, biochemistry, enzyme activities and serum cortisol were compared statistically using the non-parametric Mann–Whitney U-test considering the groups of seronegative* versus* *B. besnoiti* seropositive dairy cattle, and seronegative or asymptomatic seropositive* versus* clinically affected animals. The Mann–Whitney U-test revealed that the distribution of lymphocytes and granulocytes was significantly different in seronegative and seropositive cows (*P* = 0.022). In contrast, none of the other parameters showed a significant difference between the considered groups of animals.

The effect of *Besnoitia* infection on hematological and biochemical parameters, enzyme activities and serum cortisol levels was subsequently considered using GLMs. The interactions between *Besnoitia* infection, “number of parturitions” and/or “phase of lactation” were also considered to take into account both the influence of the parasite infection and the physiological status of the cows on the considered laboratory parameters. Final models in which the results on the selected parameters were associated with *B. besnoiti* infection are reported in Table 2; final models in which only the variables “number of parturitions” or “phase of lactation” were statistically associated to the response variable are reported in Additional file 3: Table S3. Only results related to *B. besnoiti* infection are discussed, since those related merely to the physiological status of the cows are not relevant to this study. The data of each full model with reference to backward selection procedure are summarized in Additional file 4: Tables S4.

**Table 2:** Results of the significative variables in the generalized linear models analysis regarding the variation in laboratory parameters according to *B. besnoiti* infection (seropositivity or clinical signs), number of parturitions and phase of lactation

Response Variable	Predictor	Category	Mean (SD)	Min–Max	β^a^	Standard error of coefficients	Wald Chi-square	Odds ratio (95% confidence interval)	*P* value	Akaike information criterion
Lymphocytes	Seropositivity to *B. besnoiti*	Seronegative	53.52 (13.69)	11.00–97.00	0			1		80.636
		Seropositive	45.78 (18.53)	12.00–88.00	− 0.08	0.03	6.30	0.93 (0.87–0.98)	0.012	
	Number of parturitions^b^	Primiparous	48.67 (16.97)	12.00–97.00	0			1		
		Multiparous	51.19 (16.38)	11.00–88.00	− 0.022	0.410	0.284	0.978 (0.903–1.060)	0.594	
	Seropositivity to *B. besnoiti* × Number of parturitions^b^	Seronegative Primiparous	54.47 (14.36)	25.00–97.00	0			1		
		Seronegative Multiparous	52.29 (13.83)	11.00–81.00	− 0.02	0.04	0.28	0.98 (0.90–1.06)	0.594	
		Seropositive Primiparous	37.81 (16.51)	12.00–65.00	− 0.17	0.05	11.92	0.85 (0.77–0.93)	0.001	
		Seropositive Multiparous	50.14 (18.70)	15.00–88.00	− 0.04	0.04	1.14	0.96 (0.88–1.04)	0.286	
	Clinical signs of besnoitiosis	Seronegative or asymptomatic seropositive cows	51.70 (16.12)	11–97	0			1		64.738
		Clinically affected	47.88 (18.15)	12–86	− 0.15	0.05	7.26	0.86 (0.78–0.96)	0.007	
	Number of parturitions^b^	Primiparous	48.67 (16.97)	12.00–97.00	0			1		
		Multiparous	51.19 (16.38)	11.00–88.00	− 0.146	0.0543	8.171	1.243 (1.071–1.444)	0.325	
	Clinical signs of besnoitiosis × Number of parturitions^b^	Seronegative or asymptomatic seropositive cows Primiparous	53.39 (15.34)	21–97	0			1		
		Seronegative or asymptomatic seropositive cows Multiparous	49.86 (17.43)	11–88	− 0.03	0.04	0.80	0.96 (0.89–1.04)	0.370	
		Clinically affected Primiparous	38.75 (17.98)	12–65	− 0.15	0.05	7.26	0.86 (0.78–0.96)	0.007	
		Clinically affected Multiparous	57.00 (13.55)	29–86	0.4	0.05	0.44	1.04 (0.93–1.15)	0.506	
Granulocytes	Seropositivity to *B. besnoiti*	Seronegative	46.48 (13.69)	3.00–89.00	0			1		80.636
		Seropositive	54.22 (18.53)	12.00–88.00	0.08	0.03	6.30	1.08 (1.02–1.15)	0.012	
	Number of parturitions^b^	Primiparous	51.33 (16.97)	3.00–88.00	0			1		
		Multiparous	48.81 (16.38)	12.0–89.00	0.022	0.410	0.284	1.022 (0.943–1.107)	0.594	
	Seropositivity to *B. besnoiti* × Number of parturitions	Seronegative Primiparous	45.53 (14.36)	3.00–65.00	0			1		
		Seronegative Multiparous	47.71 (13.83)	19.00–89.00	0.02	0.04	0.28	1.02 (0.94–1.12)	0.594	
		Seropositive Primiparous	62.19 (16.51)	35.00–88.00	0.17	0.05	11.92	1.18 (1.07–1.29)	0.001	
		Seropositive Multiparous	49.86 (18.70)	12.00–85.00	0.04	0.04	1.14	1.04 (0.96–1.13)	0.286	
	Clinical signs of besnoitiosis	Seronegative or asymptomatic seropositive cows	48.30 (16.12)	3–89	0			1		64.738
		Clinically affected	52.12 (18.15)	14–88	0.15	0.05	7.26	1.16 (1.04–1.29)	0.007	
	Number of parturitions^b^	Primiparous	51.33 (16.97)	3.00–88.00	0			1		
		Multiparous	48.81 (16.38)	12.0–89.00	0.146	0.0543	8.171	1.158 (1.041–1.288)	0.325	
	Clinical signs of besnoitiosis × Number of parturitions^b^	Seronegative or asymptomatic seropositive cows Primiparous	46.61 (15.34)	3–79	0			1		
		Seronegative or asymptomatic seropositive cows Multiparous	50.14 (17.43)	12–89	− 0.03	0.04	0.80	1.04 (0.96–1.12)	0.370	
		Clinically affected Primiparous	61.25 (17.98)	35–88	− 0.15	0.05	7.26	1.16 (1.04–1.29)	0.007	
		Clinically affected Multiparous	43.00 (13.55)	14–71	0.4	0.05	0.44	0.96 (0.87–1.07)	0.506	
Total proteins	Seropositivity to *B. besnoiti*	Seronegative	8.73 (1.11)	6.30–11.50	0			1		304.313
		Seropositive	8.34 (1.04)	5.60–10.50	− 0.42	0.20	4.30	0.66 (0.44–0.98)	0.038	
	Phase of lactation^c^	Early	8.40 (0.98)a	6.30–10.20	0			1		
		Mid	8.38 (1.06)a	5.60–11.00	− 0.02	0.26	0.00	0.98 (0.59–1.61)	0.930	
		Late	9.13 (1.02)	7.40–11.50	0.72	0.28	6.84	2.06 (1.19–3.54)	0.009	
		Dry	8.27 (1.26)a	6.30–10.50	− 0.13	0.35	0.13	0.88 (0.44–1.76)	0.717	
Albumin	Seropositivity to *B. besnoiti*	Seronegative	2.96 (0.52)	2.00–4.20	0			1		153.895
		Seropositive	3.12 (0.60)	2.00–4.70	0.23	0.09	5.75	1.26 (1.04–1.52)	0.017	
	Phase of lactation^c^	Early	2.99 (0.50)a	2.1–3.9	0			1		
		Mid	2.88 (0.42)a	2.00–4.00	− 0.11	0.12	0.86	0.89 (0.70–1.14)	0.354	
		Late	2.98 (0.65)a	2.00–4.70	− 0.00	0.13	0.00	0.99 (0.76–1.29)	0.951	
		Dry	3.68 (0.34)	2.90–4.20	0.69	0.17	16.60	2.00 (1.43–2.79)	0.000	
Globulin	Seropositivity to *B. besnoiti*	Seronegative	5.78 (1.34)	2.90–8.90	0			1		326.879
		Seropositive	5.22 (1.10)	3.20–7.70	− 0.65	0.22	8.21	0.52 (0.34–0.81)	0.004	
	Phase of lactation^c^	Early	5.41 (1.14)a	3.80–8.00	0			1		
		Mid	5.50 (1.15)a	3.60–8.70	0.09	0.29	0.09	1.09 (0.62–1.95)	0.754	
		Late	6.14 (1.27)	4.40–8.90	0.73	0.31	5.36	2.08 (1.12–3.86)	0.021	
		Dry	4.59 (1.35)	2.90–7.60	− 0.82	0.40	4.11	0.44 (0.19–0.97)	0.043	
A/G ratio	Seropositivity to *B. besnoiti*	Seronegative	0.56 (0.22)	0.26–1.28	0			1		42.458
		Seropositive	0.63 (0.21)	0.31–1.19	0.10	0.04	7.76	1.11 (1.03–1.19)	0.005	
	Phase of lactation^c^	Early	0.58 (0.19)a	0.27–1.00	0			1		
		Mid	0.55 (0.16)a	0.26–0.95	− 0.35	0.05	0.53	0.97 (0.88–1.06)	0.466	
		Late	0.52 (0.21)a	0.29–1.04	− 0.65	0.05	1.59	0.94 (0.85–1.04)	0.207	
		Dry	0.87 (0.26)	0.38–1.27	0.28	0.06	18.74	1.33 (1.17–1.51)	0.000	
GLDH	Clinical signs of besnoitiosis	Seronegative or asymptomatic seropositive cows	31.22 (48.68)	6.4–367	0			1		33.850
		Clinically affected	39.86 (37.10)	0.00–137.7	0.14	0.07	4.51	1.15 (1.01–1.31)	0.034	
	Number of parturitions^b^	Primiparous	37.72 (36.78)	0.00–163.9	0.14	0.06	6.04	1.15 (1.03–1.29	0.014	
		Multiparous	23.20 (18.64)	6.4–82	0			1		

Descriptive statistics of the considered parameters were also included

Mean (SD) values of each parameter per each phase of lactation followed by lowercase letter ‘a’ are statistically different from other values without lowercase letter ‘a’ at *P* value < 0.05 (generalized linear models analysis, pairwise comparison), while they are not statistically different from each other (*P* value > 0.05, GLM, pairwise comparison)

^a^Coefficient

^b^The number of parturitions was classified as follows: Primiparous  =  one parturition, Multiparous = two or more parturitions

^c^The phase of lactation was classified as follows: Early = 0–120 days, Mid = 121–250 days, Late = 251–305 days, and Dry

Regarding hematological parameters, only the variables lymphocytes and granulocytes were influenced by both *B. besnoiti* seropositivity and clinical disease. The percentage of lymphocytes was evidenced to be lower in seropositive (all values presented as the mean ± SD; 45.78 ± 18.53%, odds ratio [OR] 0.93) than in seronegative cows (53.52 ± 13.69%). Also, the percentage of granulocytes was 1.08-fold higher in seropositive cattle than in seronegative cattle (54.22 ± 18.53* vs* 46.48 ± 13.69%, respectively). In addition, the interaction between *B. besnoiti* seropositivity and the number of parturitions was statistically associated to the alterations in the leukocyte differential, i.e. the percentage of lymphocytes (37.81 ± 16.51, OR 0.85) was lower and the percentage of granulocytes (62.19 ± 16.51%, OR 1.18) was higher in seropositive primiparous cows than in seropositive multiparous cows and their respective seronegative counterparts. Similarly, clinically affected cows evidenced lower percentages of lymphocytes (47.88 ± 18.15%, OR 0.86) and higher percentages of granulocytes (52.12 ± 18.15%, OR 1.04) when compared to seronegative or asymptomatic seropositive cows (51.70 ± 16.12 and 48.30 ± 16.12%, respectively). Also, in this case, the interaction between clinical signs of besnoitiosis and the number of parturitions was associated with lower and higher percentage values of lymphocytes (38.75 ± 17.98%, OR 0.86) and granulocytes (61.25 ± 17.98%, OR  1.16), respectively, in clinically affected primiparous cows, when compared to multiparous animals with clinical signs of chronic besnoitiosis and seronegative or asymptomatic seropositive animals (Table 2).

Regarding biochemical analytes, alterations in serum protein values were associated with *B. besnoiti* serological status and the lactation phase. Parasite infection was a predictor of lower values of total protein (8.34 ± 1.04 g/dl, OR 0.66) and globulin (5.22 ± 1.10 g/dl, OR  0.52) in seropositive cows, in comparison to seronegative animals (8.73 ± 1.11 and 5.78 ± 1.34 g/dl, respectively). On the contrary, higher values of albumin (3.12 ± 0.60 g/dl, OR 1.26) and A/G ratio (0.63 ± 0.21, OR 1.11) were evidenced in seropositive cows compared to seronegative ones (2.96 ± 0.52 g/dl and 0.56 ± 0.22, respectively). Regarding the lactation phase, higher total protein values (9.13 ± 1.02 g/dl, OR  2.06) were evidenced in cows in the late phase of lactation, whereas dry cows showed a higher probability to have higher values for both albumin (3.68 ± 0.34 g/dl, OR 2.00) and A/G ratio (0.87 ± 0.26, OR 1.33). Furthermore, cows in the late phase of lactation and dry animals had a higher probability to show higher and lower globulin values (6.15 ± 1.27, OR 2.08 and 4.59 ± 1.35 g/dl, OR  0.44, respectively) than cows of other lactation categories (Table 2).

Among the enzyme activity parameters, the presence of clinical signs of besnoitiosis and the number of parturitions were associated to alterations in GLDH values. Indeed, clinically affected animals had an enhanced probability to have higher mean values of GLDH (39.86 ± 37.10 U/l, OR 1.15) when compared to seronegative or asymptomatic seropositive cows (31.22 ± 48.68 U/l). Similarly, primiparous cows (37.72 ± 36.78 U/l, OR 1.15) had an enhanced probability to have higher GLDH values than multiparous animals (23.20 ± 18.64 U/l).

## Discussion

The study provides data on laboratory parameters, including hematology, biochemistry, enzyme activity and cortisol determination, in *B. besnoiti* naturally infected cows from an endemically affected dairy cattle herd in Italy. Results were analyzed taking into consideration both the serological status for *B. besnoiti* (seronegative and seropositive cows) and the presence of clinical chronic bovine besnoitiosis (seronegative or asymptomatic seropositive cows and clinically affected cows). The influence of the number of parturitions (primiparous and multiparous cows) and the phase of lactation (early, mid, late and dry) on laboratory parameters were also considered. Finally, the two-way interactions between *Besnoitia* seropositivity or clinical disease, number of parturitions and phase of lactation were evaluated.

### Hematology

Mean RBC counts were at the lower limit of the reference range in all sampled animals. However, it should be noted that of the 107 cows sampled for blood parameters, 95 were lactating at the time of sampling, and lactating cows normally consistently have lower RBC counts than non-lactating cows [20]. A few authors demonstrated the occurrence of anemia in both beef cattle [9] and goats [13] with besnoitiosis, probably caused by the chronic inflammatory state.

An important alteration was evidenced in WBC counts. Even though the mean number of WBC fell within the normal range in all considered groups of animals, *B. besnoiti* infection caused an alteration in the leukocyte differential, with a higher percentage of granulocytes and a lower percentage of lymphocytes in seropositive and clinically affected cows. The same alteration was influenced by the number of parturitions, and was enhanced for seropositive and clinically affected animals in the group of primiparous cows. Many seropositive animals (69.6%), particularly primiparous cows (87.5%), presented this alteration in the leukocyte differential. Indeed, a significative interaction was evidenced between parasite infection and physiological status of the cows: this alteration in the leukocyte differential, with higher values of granulocytes and lower values of lymphocytes, was influenced not only by *B. besnoiti* seropositivity or clinical disease, but also by the physiological status of the cows. This finding suggests that the effects of the parasite infection may be enhanced in primiparous cows if compared to multiparous cows, probably due to a higher level of stress and immunosuppression in younger animals.

Langenmayer et al. [9] reported leukopenia in the acute phase of the disease followed by higher WBC concentration values after seroconversion in beef cattle. Two other studies evidenced leukocytosis in cattle infected with *B. besnoiti*: Dubey et al. [11] also reported a left-shift neutrophilia in a clinically affected bull, whereas Nieto-Rodríguez et al. [10] detected lymphocytosis in some infected beef animals. Nazifi et al. [13] evidenced that, compared to normal goats, WBC and neutrophils were significantly higher and lymphocytes were significantly lower in goats with clinical besnoitiosis.

It is recognized that in adult cattle lymphocytes remain the dominant cell type, with a neutrophil-to-lymphocyte ratio of approximately 1:2. A shift in this ratio (stress leukogram) may be due to several causes: inflammatory granulocytosis is reported in viral, bacterial, protozoal, parasitic and fungal infections, whereas stress, infections, immune suppression, also related to corticosteroids, are among the causes of lymphopenia [21]. Besides, the aggregation of lymphocytes around *Besnoitia* cysts in affected organs could also induce lymphopenia [13].

In this context, it should be emphasized that the farm is officially free of tuberculosis, brucellosis and leucosis. All of the animals in the herd are vaccinated against Bovine Viral Diarrhea Virus, and the farm adheres to the management plan for the control of infectious bovine rhinotracheitis and paratuberculosis. Further, after the diagnosis of some cases of Q fever also causing abortions in the herd 1 year earlier, all the animals were vaccinated against *Coxiella burnetii*. Moreover, it is noteworthy to emphasize that a form of generalized demodectic mange in two dairy cows co-infected with *B. besnoiti* was diagnosed and that *Demodex bovis* mites could also affect other animals, causing subclinical forms of the disease and possibly contributing to the detected alterations in WBC parameters [19]. Therefore, the observed alteration in the leukocyte differential could be a predictor of the parasite infection, suggesting the need to include bovine besnoitiosis in the differential diagnoses along with other diseases.

Any relevant alteration was not detected in platelet indexes, with only a slight reduction in the mean values of PLT counts, MPV, Pct and PDW in seropositive and clinically affected animals, suggesting that slight thrombocytopenia may indicate the presence of an inflammatory response. It is possible that the increase in platelet consumption for their destruction during the chronic inflammatory process due to the parasite infection may be the factors determining these findings [22]. However, considering that none of the previous studies investigated these parameters, it is not possible to draw significant conclusions.

### Biochemistry and enzyme activity

Serum total proteins were elevated in all cows, with a slightly higher value in seronegative animals; the parasite infection caused lower mean values of total proteins in seropositive animals. Also, a higher level of albumin and a lower level of globulin were associated with *B. besnoiti* infection, as detected in seropositive cows. Langenmayer et al. [9] reported higher values of total proteins, albumin and globulin in seropositive cattle, and some other animals with clinical besnoitiosis evidenced high levels of total proteins and albumin [10]; similar findings were also shown in clinically infected goats [14]. Moderate hypoalbuminemia and mild hyperglobulinemia were reported during the acute phase in a clinically affected bull [11]. However, it should be considered that these results refer to studies performed on beef cattle. Alshehabat et al. [12] reported a slightly lower value in total proteins with a higher value of albumin in *B. besnoiti* infected dairy cows.

Regarding total proteins, it can not be excluded that seropositive cows may manifest edematous phenomena associated with hypoproteinemia conditions that are not detectable at the clinical examination due to the infection in these animals being subclinical. Higher mean values of albumin in seropositive animals could indicate slight dehydration; instead, clinically affected animals showed a slightly lower albumin concentration when compared to the other groups of cows, probably due to inflammation (as albumin acts as a negative acute phase protein) and negative energy balance. In any case, mean values of albumin were at the lower limit of the range for all animals. The lower values of globulin in seropositive animals, even with elevated mean values in all the animals, could be due to immunosuppression, taking into consideration the higher levels of serum cortisol and the lower values of lymphocytes [23]. Moreover, it was evidenced that also the phase of lactation influenced serum protein parameters, reflecting the physiological changes occurring during the lactation period.

The higher values of total bilirubin, particularly in seropositive and clinically affected cows, evidenced also in beef cattle by Langenmayer et al. [9], could be related to both prehepatic icterus due to hemorrhages, and anorexia, sickness, and debilitation.

No alterations were detected in mean Ca, P and Mg concentrations, with all values falling within the reference ranges and no difference between seronegative and seropositive cows. However, it should be considered that blood concentrations of minerals are influenced by physiological factors, such as food intake and hydration, and can also change daily [9].

Concerning metabolites and ketones, no difference was underlined between seronegative, seropositive and clinically affected cows. However, if considering all the animals, BOHB and lactate were both slightly higher, indicating a reduced energy balance in milk-producing dairy cows, whereas NEFA values fell within the reference range.

Regarding enzyme activities, no relevant alteration was detected for AST, CK and LDH in *B. besnoiti* seropositive and clinically affected animals. Inconsistent results were also reported in previous studies [9, 12]. AST values were similar in all the considered groups of animals; CK showed a higher activity in seronegative animals, but it should be considered that some animals had very high values, distorting the mean results. According to Langenmayer et al. [9], the effect of tissue cysts on muscle fibers can be very low, leading to only slight and undetectable release of muscle enzymes. LDH mean value was slightly higher in clinically affected animals compared to seronegative ones; this alteration could be associated with tissue damage. GLDH was recorded at levels that were slightly higher than the upper limit of the reference range in all animals, with a higher mean value in clinically affected animals and, particularly, in primiparous cows; these findings could be due to the metabolic condition of the cows.

### Cortisol determination

Higher cortisol mean values were detected in seropositive and clinically affected cows when compared to seronegative animals, although the difference was not statistically significant.

Mormède et al. [15] reported that mean baseline cortisol values in cattle are typically lower than 10 ng/ml, with recent studies often reporting values of < 5 ng/mL. In the present study, the mean values of cortisol were quite high for all the herd animals: this finding could be related to poor farm management practices that were evidenced during the visits to the farm. Although statistical analysis did not show any relation between *B. besnoiti* infection and cortisol levels, it is hypothesized that, among other physiological, pathological and farm-related factors, higher values of cortisol in *B. besnoiti* seropositive and clinically affected animals may also be due to the stress related to the disease; indeed, bovine besnoitiosis is a chronic and debilitating disease, compromising animal welfare. In the same farm, a decrease in certain productive parameters (i.e. daily kilograms of milk produced, percentage of fat and protein in the milk and mature equivalent milk yield) was previously evidenced in cows showing tissue cysts in the skin, which could be correlated to the debilitation caused by the chronic phase of the disease [5]. The detection of *D. bovis* infection in two cows, seropositive for *B. besnoiti*, provides support for this hypothesis: it is known [19] that these mites develop a heavy infection mainly in dairy cows suffering from stress, and that the occurrence of bovine demodicosis seems to be associated with debilitating factors or receptive physiological states of the animal, such as pregnancy or lactation. Since bovine besnoitiosis is a chronic and debilitating disease, its role in determining an immune imbalance, possibly leading to other infections, including *D. bovis* [19], should not be neglected. Stress could be a consequence of *B. besnoiti* infection, but it has also been experimentally demonstrated that immunodepression related to corticosteroids could facilitate parasite proliferation [17]. To the best of the authors’ knowledge, this is the first study investigating cortisol levels in *B. besnoiti* seropositive and clinically affected cattle. Although the results are only preliminary, it is suggested that *B. besnoiti* may cause a higher value of cortisol and then stress in infected animals: the consequences for animal welfare should not be neglected. Besides, the effect of cortisol on both the disease onset and progression should be considered. However, also considering the lack of statistical significance, the association between *B. besnoiti* infection and cortisol levels could not be demonstrated, and this hypothesis should be further investigated.

## Conclusions

The study provides data on alterations in laboratory parameters in *B. besnoiti* seropositive and clinically affected cows from an endemically infected dairy herd in Italy. Only mild changes were found in hematology, biochemistry, and enzyme activity parameters. *Besnoitia besnoiti* infection seemed to be associated with an alteration in the leukocyte differential, characterized by a higher percentage of the granulocytes and a lower value of the lymphocytes population, particularly in primiparous cows. Considering biochemistry and enzyme activity parameters, the parasite infection caused alterations in serum protein parameters in seropositive animals and in GLDH values in clinically affected ones. Although not statistically significant, the higher cortisol levels in seropositive and clinically affected animals may suggest that bovine besnoitiosis could be related to stress in infected cows: this may lead to consequences not only on animal welfare but also on the disease onset and progression. However, laboratory values are influenced by both physiological and pathological factors. Besides, it should be considered that farm-related factors could influence results on investigated animals. Further studies involving a higher number of animals from different farms, including seropositive cattle and clinically affected cows in both acute and chronic phase of the disease, could help to determine the role of *B. besnoiti* in alterations in laboratory parameters, and to aid veterinarians in the diagnosis of bovine besnoitiosis to optimize a control plan for the disease in the infected herds.

## Supplementary Information

**Additional file 1: Table S1.** Descriptive statistics (mean, standard deviation, minimum and maximum) of hematological, biochemical and enzyme activities analyses, and cortisol determination sorted by the number of parturitions and the serological and clinical status of cows in a dairy cattle herd endemically infected by bovine besnoitiosis.

**Additional file 2: Table S2.** Descriptive statistics (mean, standard deviation, minimum and maximum) of hematological, biochemical and enzyme activities analyses, and cortisol determination sorted by the lactation phase and the serological and clinical status of cows in a dairy cattle herd endemically infected by bovine besnoitiosis.

**Additional file 3: Table S3.** Results of the significative variables in the GLM analysis regarding the variation on laboratory parameters according to number of parturitions and phase of lactation. Descriptive statistics (mean, standard deviation, minimum and maximum) of the considered parameters were also included.

**Additional file 4: Tables S4.** Selection of final model by backward elimination according to the best Akaike's information criterion (AIC). AIC values were provided for the full models and for each step corresponding to the removal of the least statistically significant variable, until the final model was obtained.

## Data Availability

The datasets used and/or analyzed during the current study are available from the corresponding author on reasonable request.
