# Supplementary material for: Exploring alterations in hematological and biochemical parameters, enzyme activities and serum cortisol in Besnoitia besnoiti naturally infected dairy cattle
Source: Parasit Vectors. 2021 Mar 15;14:154. doi: 10.1186/s13071-021-04626-4 (PMC7962361; doi:10.1186/s13071-021-04626-4)
Supplement: Supplementary file 1 — Additional file 1: Table S1. Descriptive statistics (mean, standard deviation, minimum and maximum) of hematological, biochemical and enzyme activities analyses, and cortisol determination sorted by the number of parturitions and the serological and clinical status of cows in a dairy cattle herd endemically infected by bovine besnoitiosis. [file 13071_2021_4626_MOESM1_ESM.docx]

|  | **Parameters** | **Number of Parturitions** |  | **Seronegative** | | | |  | **Seropositive** | | | | | | | |
| --- | --- | --- | --- | --- | --- | --- | --- | --- | --- | --- | --- | --- | --- | --- | --- | --- |
|  |  |  |  |  |  |  |  |  | **Overall** | | | | **Clinically affected** | | | |
|  |  |  |  | **Mean** | **SD** | **Min** | **Max** |  | **Mean** | **SD** | **Min** | **Max** | **Mean** | **SD** | **Min** | **Max** |
| **Hematology** | **RBC** | Primiparous |  | 6.31 | 0.56 | 5.28 | 7.55 |  | 6.47 | 0.55 | 5.18 | 7.02 | 6.35 | 0.60 | 5.18 | 7.02 |
|  |  | Multiparous |  | 5.76 | 0.63 | 4.67 | 7.23 |  | 5.97 | 0.68 | 4.80 | 7.14 | 5.81 | 0.59 | 4.80 | 7.02 |
|  |  | Total |  | 6.04 | 0.65 | 4.67 | 7.55 |  | 6.14 | 0.68 | 4.80 | 7.14 | 6.06 | 0.64 | 4.80 | 7.02 |
|  | **Hb** | Primiparous |  | 12.79 | 1.71 | 8.86 | 15.88 |  | 12.42 | 1.06 | 10.92 | 14.89 | 12.25 | 0.83 | 10.92 | 13.86 |
|  |  | Multiparous |  | 12.13 | 1.38 | 9.63 | 14.71 |  | 12.00 | 2.05 | 3.81 | 14.63 | 11.81 | 2.62 | 3.81 | 14.45 |
|  |  | Total |  | 12.47 | 1.58 | 8.86 | 15.88 |  | 12.15 | 1.76 | 3.81 | 14.89 | 12.01 | 1.98 | 3.81 | 14.45 |
|  | **Ht** | Primiparous |  | 27.35 | 2.40 | 21.50 | 31.80 |  | 27.92 | 1.67 | 24.00 | 30.20 | 27.37 | 1.65 | 24.00 | 30.00 |
|  |  | Multiparous |  | 26.01 | 2.57 | 20.70 | 31.00 |  | 26.87 | 3.03 | 22.20 | 32.40 | 26.02 | 2.58 | 22.20 | 30.80 |
|  |  | Total |  | 26.70 | 2.56 | 20.70 | 31.80 |  | 27.24 | 2.66 | 22.20 | 32.40 | 26.65 | 2.27 | 22.20 | 30.80 |
|  | **MCV** | Primiparous |  | 43.40 | 3.04 | 37.00 | 49.00 |  | 43.44 | 3.16 | 39.00 | 49.00 | 43.38 | 3.53 | 39.00 | 49.00 |
|  |  | Multiparous |  | 45.39 | 3.51 | 40.00 | 56.00 |  | 45.21 | 3.03 | 38.00 | 50.00 | 45.00 | 3.21 | 39.00 | 50.00 |
|  |  | Total |  | 44.36 | 3.40 | 37.00 | 56.00 |  | 44.58 | 3.16 | 38.00 | 50.00 | 44.25 | 3.40 | 39.00 | 50.00 |
|  | **MCH** | Primiparous |  | 20.38 | 2.99 | 15.55 | 25.84 |  | 19.37 | 2.55 | 15.69 | 24.36 | 19.47 | 2.31 | 15.69 | 24.36 |
|  |  | Multiparous |  | 21.20 | 2.62 | 16.12 | 27.48 |  | 20.19 | 3.45 | 7.62 | 24.96 | 20.28 | 4.17 | 7.62 | 24.96 |
|  |  | Total |  | 20.78 | 2.83 | 15.55 | 27.48 |  | 19.89 | 3.15 | 7.62 | 24.96 | 19.90 | 3.40 | 7.62 | 24.96 |
|  | **MCHC** | Primiparous |  | 46.88 | 5.91 | 36.77 | 62.35 |  | 44.64 | 4.75 | 37.38 | 54.35 | 44.87 | 3.39 | 39.45 | 52.51 |
|  |  | Multiparous |  | 46.82 | 5.13 | 38.38 | 56.51 |  | 44.73 | 7.23 | 17.17 | 56.71 | 45.22 | 9.26 | 17.17 | 56.71 |
|  |  | Total |  | 46.85 | 5.50 | 36.77 | 62.35 |  | 44.70 | 6.40 | 17.17 | 56.71 | 45.05 | 7.04 | 17.17 | 56.71 |
|  | **RDW** | Primiparous |  | 15.38 | 0.94 | 14.00 | 17.30 |  | 15.46 | 0.69 | 13.90 | 16.50 | 15.42 | 0.79 | 13.90 | 16.50 |
|  |  | Multiparous |  | 14.98 | 0.96 | 13.40 | 17.00 |  | 15.10 | 0.83 | 13.80 | 16.90 | 14.95 | 0.83 | 13.90 | 16.90 |
|  |  | Total |  | 15.19 | 0.96 | 13.40 | 17.30 |  | 15.22 | 0.79 | 13.80 | 16.90 | 15.17 | 0.83 | 13.90 | 16.90 |
|  | **WBC** | Primiparous |  | 10.38 | 8.02 | 2.70 | 48.10 |  | 10.19 | 3.71 | 6.80 | 17.70 | 10.57 | 4.00 | 6.80 | 17.70 |
|  |  | Multiparous |  | 8.36 | 4.13 | 4.20 | 26.20 |  | 9.57 | 7.83 | 4.70 | 37.20 | 9.06 | 7.73 | 4.70 | 35.80 |
|  |  | Total |  | 9.40 | 6.46 | 2.70 | 48.10 |  | 9.79 | 6.62 | 4.70 | 37.20 | 9.76 | 6.22 | 4.70 | 35.80 |
|  | **Lymphocytes** | Primiparous |  | 54.47 | 14.36 | 25.00 | 97.00 |  | 37.81 | 16.51 | 12.00 | 65.00 | 43.23 | 23.61 | 12.00 | 97.00 |
|  |  | Multiparous |  | 52.29 | 13.83 | 11.00 | 81.00 |  | 50.14 | 18.70 | 15.00 | 88.00 | 52.67 | 16.93 | 20.00 | 86.00 |
|  |  | Total |  | 53.41 | 14.02 | 11.00 | 97.00 |  | 45.76 | 18.73 | 12.00 | 88.00 | 48.29 | 20.48 | 12.00 | 97.00 |
|  | **Granulocytes** | Primiparous |  | 45.53 | 14.36 | 3.00 | 75.00 |  | 62.19 | 16.51 | 35.00 | 88.00 | 56.77 | 23.61 | 3.00 | 88.00 |
|  |  | Multiparous |  | 47.71 | 13.83 | 19.00 | 89.00 |  | 49.86 | 18.70 | 12.00 | 85.00 | 47.33 | 16.93 | 14.00 | 80.00 |
|  |  | Total |  | 46.59 | 14.02 | 3.00 | 89.00 |  | 54.24 | 18.73 | 12.00 | 88.00 | 51.71 | 20.48 | 3.00 | 88.00 |
|  | **PLT** | Primiparous |  | 291.10 | 127.36 | 17.10 | 488.00 |  | 319.63 | 135.95 | 90.00 | 557.00 | 319.23 | 140.15 | 90.00 | 557.00 |
|  |  | Multiparous |  | 326.86 | 97.84 | 102.00 | 490.00 |  | 274.10 | 81.11 | 115.00 | 449.00 | 272.00 | 88.98 | 115.00 | 449.00 |
|  |  | Total |  | 308.36 | 114.50 | 17.10 | 490.00 |  | 290.29 | 104.76 | 90.00 | 557.00 | 293.93 | 115.80 | 90.00 | 557.00 |
|  | **MPV** | Primiparous |  | 18.19 | 50.39 | 8.60 | 285.00 |  | 8.83 | 0.32 | 8.30 | 9.20 | 8.78 | 0.34 | 8.30 | 9.20 |
|  |  | Multiparous |  | 8.75 | 0.33 | 8.20 | 9.50 |  | 8.80 | 0.30 | 8.10 | 9.50 | 8.79 | 0.26 | 8.50 | 9.40 |
|  |  | Total |  | 13.63 | 36.26 | 8.20 | 285.00 |  | 8.81 | 0.31 | 8.10 | 9.50 | 8.79 | 0.30 | 8.30 | 9.40 |
|  | **Pct** | Primiparous |  | 0.27 | 0.10 | 0.07 | 0.42 |  | 0.28 | 0.12 | 0.08 | 0.47 | 0.28 | 0.12 | 0.08 | 0.47 |
|  |  | Multiparous |  | 0.29 | 0.08 | 0.08 | 0.43 |  | 0.24 | 0.07 | 0.10 | 0.38 | 0.23 | 0.08 | 0.10 | 0.38 |
|  |  | Total |  | 0.28 | 0.09 | 0.07 | 0.43 |  | 0.25 | 0.09 | 0.08 | 0.47 | 0.25 | 0.10 | 0.08 | 0.47 |
|  | **PDW** | Primiparous |  | 11.93 | 0.90 | 10.40 | 14.30 |  | 11.73 | 1.25 | 9.70 | 14.20 | 11.61 | 1.37 | 9.70 | 14.20 |
|  |  | Multiparous |  | 15.00 | 19.23 | 9.70 | 113.00 |  | 11.59 | 0.95 | 9.80 | 13.10 | 11.59 | 1.00 | 10.30 | 13.10 |
|  |  | Total |  | 13.41 | 13.34 | 9.70 | 113.00 |  | 11.64 | 1.05 | 9.70 | 14.20 | 11.60 | 1.17 | 9.70 | 14.20 |
| **Biochemistry** | **Total protein** | Primiparous |  | 8.69 | 1.04 | 6.30 | 11.50 |  | 8.26 | 0.88 | 5.60 | 9.30 | 8.15 | 0.92 | 5.60 | 9.10 |
|  |  | Multiparous |  | 8.86 | 1.21 | 6.60 | 11.00 |  | 8.40 | 1.15 | 6.30 | 10.50 | 8.39 | 1.16 | 6.30 | 10.50 |
|  |  | Total |  | 8.77 | 1.12 | 6.30 | 11.50 |  | 8.35 | 1.05 | 5.60 | 10.50 | 8.28 | 1.04 | 5.60 | 10.50 |
|  | **Albumin** | Primiparous |  | 2.95 | 0.45 | 2.40 | 3.90 |  | 3.02 | 0.51 | 2.00 | 3.70 | 2.95 | 0.53 | 2.00 | 3.70 |
|  |  | Multiparous |  | 2.88 | 0.52 | 2.00 | 3.90 |  | 3.15 | 0.63 | 2.10 | 4.70 | 2.87 | 0.52 | 2.10 | 3.90 |
|  |  | Total |  | 2.91 | 0.48 | 2.00 | 3.90 |  | 3.10 | 0.59 | 2.00 | 4.70 | 2.91 | 0.52 | 2.00 | 3.90 |
|  | **Globulin** | Primiparous |  | 5.75 | 1.19 | 3.10 | 8.90 |  | 5.24 | 0.72 | 3.60 | 6.30 | 5.20 | 0.73 | 3.60 | 6.30 |
|  |  | Multiparous |  | 5.98 | 1.45 | 2.90 | 8.70 |  | 5.25 | 1.29 | 3.20 | 7.70 | 5.51 | 1.04 | 3.80 | 6.80 |
|  |  | Total |  | 5.86 | 1.32 | 2.90 | 8.90 |  | 5.24 | 1.11 | 3.20 | 7.70 | 5.37 | 0.91 | 3.60 | 6.80 |
|  | **A/G ratio** | Primiparous |  | 0.54 | 0.18 | 0.29 | 1.03 |  | 0.59 | 0.13 | 0.41 | 0.80 | 0.58 | 0.12 | 0.42 | 0.80 |
|  |  | Multiparous |  | 0.53 | 0.23 | 0.26 | 1.28 |  | 0.65 | 0.24 | 0.31 | 1.19 | 0.54 | 0.15 | 0.37 | 0.82 |
|  |  | Total |  | 0.54 | 0.21 | 0.26 | 1.28 |  | 0.63 | 0.20 | 0.31 | 1.19 | 0.56 | 0.14 | 0.37 | 0.82 |
|  | **Total bilirubin** | Primiparous |  | 0.60 | 0.43 | 0.18 | 1.63 |  | 0.61 | 0.67 | 0.13 | 3.01 | 0.64 | 0.75 | 0.13 | 3.01 |
|  |  | Multiparous |  | 0.51 | 0.29 | 0.09 | 1.34 |  | 0.63 | 0.95 | 0.13 | 5.26 | 0.76 | 1.26 | 0.23 | 5.26 |
|  |  | Total |  | 0.56 | 0.37 | 0.09 | 1.63 |  | 0.62 | 0.85 | 0.13 | 5.26 | 0.70 | 1.04 | 0.13 | 5.26 |
|  | **Ca** | Primiparous |  | 9.71 | 0.62 | 8.00 | 10.50 |  | 9.78 | 0.83 | 7.10 | 10.80 | 9.65 | 0.89 | 7.10 | 10.80 |
|  |  | Multiparous |  | 9.30 | 0.78 | 7.00 | 10.40 |  | 9.38 | 0.62 | 7.40 | 10.40 | 9.19 | 0.66 | 7.40 | 10.00 |
|  |  | Total |  | 9.51 | 0.73 | 7.00 | 10.50 |  | 9.53 | 0.72 | 7.10 | 10.80 | 9.40 | 0.79 | 7.10 | 10.80 |
|  | **P** | Primiparous |  | 6.35 | 1.13 | 4.30 | 8.30 |  | 6.29 | 1.15 | 4.40 | 8.30 | 6.14 | 1.21 | 4.40 | 8.30 |
|  |  | Multiparous |  | 6.28 | 1.09 | 3.90 | 7.90 |  | 6.08 | 1.26 | 4.00 | 10.40 | 6.08 | 1.46 | 4.00 | 10.40 |
|  |  | Total |  | 6.32 | 1.10 | 3.90 | 8.30 |  | 6.16 | 1.21 | 4.00 | 10.40 | 6.11 | 1.33 | 4.00 | 10.40 |
|  | **Mg** | Primiparous |  | 2.17 | 0.32 | 1.38 | 2.73 |  | 2.17 | 0.34 | 1.22 | 2.64 | 2.18 | 0.36 | 1.22 | 2.64 |
|  |  | Multiparous |  | 2.12 | 0.38 | 1.42 | 3.10 |  | 2.13 | 0.36 | 1.46 | 2.93 | 2.14 | 0.36 | 1.46 | 2.65 |
|  |  | Total |  | 2.15 | 0.35 | 1.38 | 3.10 |  | 2.14 | 0.35 | 1.22 | 2.93 | 2.16 | 0.35 | 1.22 | 2.65 |
|  | **NEFA** | Primiparous |  | 0.34 | 0.24 | 0.09 | 1.05 |  | 0.33 | 0.19 | 0.12 | 0.71 | 0.32 | 0.20 | 0.12 | 0.71 |
|  |  | Multiparous |  | 0.31 | 0.25 | 0.06 | 1.02 |  | 0.34 | 0.21 | 0.09 | 0.76 | 0.32 | 0.21 | 0.14 | 0.76 |
|  |  | Total |  | 0.33 | 0.24 | 0.06 | 1.05 |  | 0.34 | 0.20 | 0.09 | 0.76 | 0.32 | 0.20 | 0.12 | 0.76 |
|  | **BOHB** | Primiparous |  | 0.62 | 0.25 | 0.29 | 1.54 |  | 0.62 | 0.20 | 0.32 | 1.06 | 0.58 | 0.17 | 0.32 | 0.98 |
|  |  | Multiparous |  | 0.66 | 0.23 | 0.32 | 1.20 |  | 0.60 | 0.31 | 0.05 | 1.48 | 0.70 | 0.39 | 0.05 | 1.48 |
|  |  | Total |  | 0.64 | 0.24 | 0.29 | 1.54 |  | 0.61 | 0.28 | 0.05 | 1.48 | 0.65 | 0.31 | 0.05 | 1.48 |
|  | **Lactate** | Primiparous |  | 2.77 | 1.01 | 1.47 | 5.23 |  | 2.87 | 1.05 | 1.10 | 5.39 | 2.96 | 1.09 | 1.10 | 5.39 |
|  |  | Multiparous |  | 2.80 | 0.78 | 1.51 | 4.50 |  | 2.77 | 0.90 | 1.21 | 4.84 | 2.66 | 0.95 | 1.21 | 4.84 |
|  |  | Total |  | 2.78 | 0.89 | 1.47 | 5.23 |  | 2.80 | 0.95 | 1.10 | 5.39 | 2.80 | 1.01 | 1.10 | 5.39 |
| **Enzymes** | **AST** | Primiparous |  | 81.07 | 40.76 | 43.00 | 252.00 |  | 73.63 | 25.77 | 49.00 | 159.00 | 74.46 | 28.89 | 49.00 | 159.00 |
|  |  | Multiparous |  | 68.00 | 24.13 | 44.00 | 171.00 |  | 73.46 | 18.56 | 47.00 | 131.00 | 74.53 | 17.65 | 48.00 | 110.00 |
|  |  | Total |  | 74.65 | 33.98 | 43.00 | 252.00 |  | 73.52 | 21.16 | 47.00 | 159.00 | 74.50 | 23.08 | 48.00 | 159.00 |
|  | **CK** | Primiparous |  | 468.21 | 1828.68 | 28.00 | 9971.00 |  | 104.94 | 31.82 | 62.00 | 183.00 | 106.69 | 32.16 | 62.00 | 183.00 |
|  |  | Multiparous |  | 153.07 | 214.93 | 57.00 | 1137.00 |  | 141.36 | 161.56 | 37.00 | 782.00 | 178.40 | 211.53 | 39.00 | 782.00 |
|  |  | Total |  | 313.40 | 1311.32 | 28.00 | 9971.00 |  | 128.11 | 130.60 | 37.00 | 782.00 | 145.11 | 158.07 | 39.00 | 782.00 |
|  | **LDH** | Primiparous |  | 912.90 | 223.61 | 434.00 | 1362.00 |  | 962.44 | 340.17 | 601.00 | 2113.00 | 975.46 | 367.74 | 601.00 | 2113.00 |
|  |  | Multiparous |  | 826.89 | 305.39 | 453.00 | 2173.00 |  | 804.18 | 171.01 | 464.00 | 1094.00 | 849.07 | 179.40 | 535.00 | 1094.00 |
|  |  | Total |  | 870.65 | 268.05 | 434.00 | 2173.00 |  | 861.73 | 254.28 | 464.00 | 2113.00 | 907.75 | 284.45 | 535.00 | 2113.00 |
|  | **GLDH** | Primiparous |  | 34.75 | 34.02 | 10.00 | 163.90 |  | 42.91 | 41.83 | 0.00 | 137.70 | 47.35 | 45.10 | 0.00 | 137.70 |
|  |  | Multiparous |  | 20.63 | 14.90 | 6.40 | 75.40 |  | 25.78 | 21.72 | 6.90 | 82.00 | 26.91 | 21.76 | 6.90 | 81.30 |
|  |  | Total |  | 27.69 | 26.98 | 6.40 | 163.90 |  | 32.01 | 31.24 | 0.00 | 137.70 | 36.40 | 35.46 | 0.00 | 137.70 |
|  | **Cortisol** | Primiparous |  | 6.12 | 4.63 | 0.77 | 19.10 |  | 8.65 | 10.09 | 1.25 | 40.41 | 8.73 | 11.14 | 1.25 | 40.41 |
|  |  | Multiparous |  | 6.96 | 7.56 | 1.02 | 35.48 |  | 8.96 | 9.30 | 0.56 | 43.86 | 8.00 | 11.62 | 0.56 | 43.86 |
|  |  | Total |  | 6.55 | 6.24 | 0.77 | 35.48 |  | 8.85 | 9.47 | 0.56 | 43.86 | 8.34 | 11.18 | 0.56 | 43.86 |

Supplementary Tab. 1. Descriptive statistics (mean, standard deviation, minimum and maximum) of hematological, biochemical and enzyme activities analyses, and cortisol determination sorted by the number of parturitions and the serological and clinical status of cows in a dairy cattle herd endemically infected by bovine besnoitiosis. The number of parturitions was classified as follows: Primiparous = one parturition, Multiparous = two or more parturitions. Serological status (seronegative or seropositive) was determined according to Western Blot results while as clinically affected cows are meant those animals with the presence of clinical signs of the disease.
