# Supplementary material for: Exploring alterations in hematological and biochemical parameters, enzyme activities and serum cortisol in Besnoitia besnoiti naturally infected dairy cattle
Source: Parasit Vectors. 2021 Mar 15;14:154. doi: 10.1186/s13071-021-04626-4 (PMC7962361; doi:10.1186/s13071-021-04626-4)
Supplement: Supplementary file 2 — Additional file 2: Table S2. Descriptive statistics (mean, standard deviation, minimum and maximum) of hematological, biochemical and enzyme activities analyses, and cortisol determination sorted by the lactation phase and the serological and clinical status of cows in a dairy cattle herd endemically infected by bovine besnoitiosis. [file 13071_2021_4626_MOESM2_ESM.docx]

|  | **Parameters** | **Lactation Phase** |  | **Seronegative** | | | |  | **Seropositive** | | | | | | | |
| --- | --- | --- | --- | --- | --- | --- | --- | --- | --- | --- | --- | --- | --- | --- | --- | --- |
|  |  |  |  |  |  |  |  |  | **Overall** | | | | **Clinically affected** | | | |
|  |  |  |  | **Mean** | **SD** | **Min-** | **Max** |  | **Mean** | **SD** | **Min-** | **Max** | **Mean** | **SD** | **Min-** | **Max** |
| **Hematology** | **RBC** | Early |  | 5.68 | 0.72 | 4.67 | 7.30 |  | 5.62 | 0.68 | 4.80 | 7.01 | 5.56 | 0.68 | 4.80 | 6.96 |
|  |  | Mid |  | 6.27 | 0.66 | 5.10 | 7.55 |  | 6.48 | 0.45 | 5.69 | 7.02 | 6.46 | 0.50 | 5.69 | 7.02 |
|  |  | Late |  | 6.19 | 0.48 | 5.32 | 6.84 |  | 6.31 | 0.48 | 5.62 | 7.14 | 6.04 | 0.34 | 5.62 | 6.65 |
|  |  | Dry |  | 6.45 | 0.98 | 5.51 | 8.53 |  | 6.14 | 1.09 | 4.93 | 7.04 | - | - | - | - |
|  |  | Total |  | 6.11 | 0.73 | 4.67 | 8.53 |  | 6.15 | 0.67 | 4.80 | 7.14 | 6.07 | 0.66 | 4.80 | 7.02 |
|  | **Hb** | Early |  | 11.94 | 1.63 | 8.86 | 14.34 |  | 11.27 | 2.50 | 3.81 | 13.83 | 10.75 | 2.89 | 3.81 | 13.83 |
|  |  | Mid |  | 12.94 | 1.48 | 9.71 | 15.88 |  | 12.36 | 1.30 | 10.30 | 14.89 | 12.57 | 1.08 | 10.30 | 14.45 |
|  |  | Late |  | 12.60 | 1.63 | 9.67 | 15.59 |  | 12.73 | 0.71 | 11.29 | 13.68 | 12.91 | 0.71 | 11.69 | 13.68 |
|  |  | Dry |  | 12.14 | 1.25 | 10.41 | 14.89 |  | 13.19 | 1.67 | 11.36 | 14.63 | - | - | - | - |
|  |  | Total |  | 12.46 | 1.55 | 8.86 | 15.88 |  | 12.18 | 1.75 | 3.81 | 14.89 | 12.04 | 2.01 | 3.81 | 14.45 |
|  | **Ht** | Early |  | 25.55 | 2.76 | 20.70 | 31.00 |  | 25.84 | 2.73 | 22.20 | 31.20 | 25.78 | 2.70 | 22.20 | 29.10 |
|  |  | Mid |  | 27.34 | 2.56 | 21.50 | 31.10 |  | 27.36 | 1.98 | 23.60 | 30.80 | 27.16 | 2.13 | 23.60 | 30.80 |
|  |  | Late |  | 26.63 | 1.97 | 23.60 | 30.40 |  | 28.54 | 2.48 | 24.70 | 32.20 | 27.12 | 1.90 | 24.70 | 29.70 |
|  |  | Dry |  | 28.01 | 2.72 | 23.90 | 31.80 |  | 29.67 | 4.65 | 24.30 | 32.40 | - | - | - | - |
|  |  | Total |  | 26.77 | 2.60 | 20.70 | 31.80 |  | 27.35 | 2.73 | 22.20 | 32.40 | 26.69 | 2.30 | 22.20 | 30.80 |
|  | **MCV** | Early |  | 45.12 | 2.85 | 40.00 | 49.00 |  | 46.07 | 2.81 | 39.00 | 50.00 | 46.44 | 3.28 | 39.00 | 50.00 |
|  |  | Mid |  | 43.80 | 3.83 | 38.00 | 56.00 |  | 42.35 | 2.37 | 38.00 | 46.00 | 42.25 | 2.38 | 39.00 | 46.00 |
|  |  | Late |  | 43.27 | 3.24 | 37.00 | 49.00 |  | 45.50 | 3.00 | 41.00 | 49.00 | 45.17 | 3.60 | 41.00 | 49.00 |
|  |  | Dry |  | 44.11 | 6.70 | 32.00 | 50.00 |  | 48.33 | 2.08 | 46.00 | 50.00 | - | - | - | - |
|  |  | Total |  | 44.08 | 3.97 | 32.00 | 56.00 |  | 44.70 | 3.22 | 38.00 | 50.00 | 44.30 | 3.45 | 39.00 | 50.00 |
|  | **MCH** | Early |  | 21.21 | 3.13 | 15.55 | 27.48 |  | 20.11 | 4.36 | 7.62 | 24.96 | 19.35 | 5.12 | 7.62 | 24.96 |
|  |  | Mid |  | 20.78 | 2.67 | 16.05 | 25.96 |  | 19.20 | 2.74 | 15.32 | 24.36 | 19.61 | 2.61 | 16.34 | 24.36 |
|  |  | Late |  | 20.47 | 3.06 | 16.12 | 25.50 |  | 20.29 | 2.11 | 16.31 | 22.87 | 21.41 | 1.41 | 18.85 | 22.87 |
|  |  | Dry |  | 19.08 | 2.69 | 14.74 | 22.98 |  | 21.61 | 1.25 | 20.79 | 23.05 | - | - | - | - |
|  |  | Total |  | 20.57 | 2.91 | 14.74 | 27.48 |  | 19.92 | 3.12 | 7.62 | 24.96 | 19.92 | 3.46 | 7.62 | 24.96 |
|  | **MCHC** | Early |  | 46.93 | 6.39 | 36.92 | 62.35 |  | 43.47 | 8.54 | 17.17 | 53.21 | 41.35 | 9.79 | 17.17 | 49.73 |
|  |  | Mid |  | 47.44 | 4.63 | 38.64 | 56.33 |  | 45.39 | 5.83 | 37.50 | 56.71 | 46.53 | 5.33 | 39.40 | 56.71 |
|  |  | Late |  | 47.42 | 5.87 | 38.38 | 59.96 |  | 44.93 | 5.01 | 37.38 | 53.29 | 47.76 | 3.64 | 44.91 | 53.29 |
|  |  | Dry |  | 43.66 | 5.59 | 35.95 | 53.69 |  | 44.64 | 2.42 | 42.01 | 46.76 | - | - | - | - |
|  |  | Total |  | 46.74 | 5.62 | 35.95 | 62.35 |  | 44.64 | 6.34 | 17.17 | 56.71 | 45.08 | 7.17 | 17.17 | 56.71 |
|  | **RDW** | Early |  | 14.94 | 0.97 | 13.40 | 17.30 |  | 14.54 | 0.67 | 13.80 | 15.90 | 14.39 | 0.56 | 13.90 | 15.70 |
|  |  | Mid |  | 15.47 | 0.94 | 13.70 | 17.20 |  | 15.70 | 0.57 | 14.70 | 16.90 | 15.86 | 0.56 | 15.00 | 16.90 |
|  |  | Late |  | 15.49 | 0.84 | 14.00 | 16.70 |  | 15.29 | 0.68 | 14.60 | 16.60 | 15.08 | 0.44 | 14.60 | 15.60 |
|  |  | Dry |  | 14.82 | 1.23 | 13.60 | 17.50 |  | 15.37 | 0.72 | 14.90 | 16.20 | - | - | - | - |
|  |  | Total |  | 15.23 | 0.99 | 13.40 | 17.50 |  | 15.22 | 0.79 | 13.80 | 16.90 | 15.20 | 0.84 | 13.90 | 16.90 |
|  | **WBC** | Early |  | 11.61 | 10.61 | 4.20 | 48.10 |  | 7.79 | 3.00 | 5.00 | 17.70 | 8.16 | 3.68 | 5.00 | 17.70 |
|  |  | Mid |  | 9.18 | 3.98 | 4.50 | 23.50 |  | 12.69 | 9.63 | 5.70 | 37.20 | 11.52 | 8.52 | 5.70 | 35.80 |
|  |  | Late |  | 7.49 | 2.59 | 2.70 | 12.90 |  | 8.36 | 2.83 | 4.70 | 14.60 | 8.38 | 3.57 | 4.70 | 14.60 |
|  |  | Dry |  | 8.58 | 2.42 | 5.00 | 12.20 |  | 6.83 | 2.19 | 5.10 | 9.30 | - | - | - | - |
|  |  | Total |  | 9.35 | 6.31 | 2.70 | 48.10 |  | 9.69 | 6.58 | 4.70 | 37.20 | 9.70 | 6.33 | 4.70 | 35.80 |
|  | **Lymphocytes** | Early |  | 57.00 | 18.66 | 22.00 | 97.00 |  | 46.36 | 16.72 | 27.00 | 88.00 | 45.56 | 13.76 | 27.00 | 64.00 |
|  |  | Mid |  | 52.80 | 10.49 | 25.00 | 68.00 |  | 50.12 | 20.93 | 12.00 | 86.00 | 50.00 | 21.20 | 12.00 | 86.00 |
|  |  | Late |  | 54.20 | 9.31 | 41.00 | 71.00 |  | 40.92 | 17.06 | 12.00 | 64.00 | 40.83 | 20.28 | 12.00 | 64.00 |
|  |  | Dry |  | 47.44 | 15.05 | 11.00 | 59.00 |  | 38.00 | 20.07 | 15.00 | 52.00 | - | - | - | - |
|  |  | Total |  | 53.52 | 13.69 | 11.00 | 97.00 |  | 45.78 | 18.53 | 12.00 | 88.00 | 46.48 | 18.46 | 12.00 | 86.00 |
|  | **Granulocytes** | Early |  | 43.00 | 18.66 | 3.00 | 78.00 |  | 53.64 | 16.72 | 12.00 | 73.00 | 54.44 | 13.76 | 36.00 | 73.00 |
|  |  | Mid |  | 47.20 | 10.49 | 32.00 | 75.00 |  | 49.88 | 20.93 | 14.00 | 88.00 | 50.00 | 21.20 | 14.00 | 88.00 |
|  |  | Late |  | 45.80 | 9.31 | 29.00 | 59.00 |  | 59.08 | 17.06 | 36.00 | 88.00 | 59.17 | 20.28 | 36.00 | 88.00 |
|  |  | Dry |  | 52.56 | 15.05 | 41.00 | 89.00 |  | 62.00 | 20.07 | 48.00 | 85.00 | - | - | - | - |
|  |  | Total |  | 46.48 | 13.69 | 3.00 | 89.00 |  | 54.22 | 18.53 | 12.00 | 88.00 | 53.52 | 18.46 | 14.00 | 88.00 |
|  | **PLT** | Early |  | 331.71 | 104.47 | 112.00 | 490.00 |  | 293.29 | 82.92 | 90.00 | 449.00 | 289.78 | 101.58 | 90.00 | 449.00 |
|  |  | Mid |  | 304.41 | 139.51 | 17.10 | 464.00 |  | 293.47 | 133.92 | 137.00 | 557.00 | 288.17 | 135.50 | 137.00 | 557.00 |
|  |  | Late |  | 302.80 | 110.95 | 75.00 | 463.00 |  | 278.25 | 95.10 | 115.00 | 453.00 | 279.33 | 87.26 | 115.00 | 355.00 |
|  |  | Dry |  | 278.33 | 85.95 | 129.00 | 426.00 |  | 247.00 | 124.74 | 112.00 | 358.00 | - | - | - | - |
|  |  | Total |  | 307.77 | 115.04 | 17.10 | 490.00 |  | 286.41 | 106.87 | 90.00 | 557.00 | 286.74 | 111.46 | 90.00 | 557.00 |
|  | **MPV** | Early |  | 8.81 | 0.38 | 8.30 | 9.50 |  | 8.66 | 0.31 | 8.10 | 9.20 | 8.69 | 0.33 | 8.30 | 9.20 |
|  |  | Mid |  | 22.70 | 61.74 | 8.20 | 285.00 |  | 8.88 | 0.28 | 8.40 | 9.40 | 8.90 | 0.30 | 8.40 | 9.40 |
|  |  | Late |  | 8.90 | 0.27 | 8.40 | 9.40 |  | 8.88 | 0.32 | 8.50 | 9.50 | 8.75 | 0.22 | 8.50 | 9.10 |
|  |  | Dry |  | 9.10 | 0.38 | 8.60 | 9.80 |  | 9.03 | 0.35 | 8.70 | 9.40 | - | - | - | - |
|  |  | Total |  | 13.43 | 35.35 | 8.20 | 285.00 |  | 8.82 | 0.32 | 8.10 | 9.50 | 8.80 | 0.30 | 8.30 | 9.40 |
|  | **Pct** | Early |  | 0.29 | 0.09 | 0.11 | 0.43 |  | 0.25 | 0.07 | 0.08 | 0.38 | 0.25 | 0.08 | 0.08 | 0.38 |
|  |  | Mid |  | 0.28 | 0.11 | 0.08 | 0.40 |  | 0.25 | 0.12 | 0.12 | 0.47 | 0.25 | 0.12 | 0.12 | 0.47 |
|  |  | Late |  | 0.27 | 0.10 | 0.07 | 0.40 |  | 0.25 | 0.08 | 0.10 | 0.40 | 0.24 | 0.07 | 0.10 | 0.31 |
|  |  | Dry |  | 0.25 | 0.09 | 0.12 | 0.42 |  | 0.22 | 0.11 | 0.11 | 0.32 | - | - | - | - |
|  |  | Total |  | 0.28 | 0.09 | 0.07 | 0.43 |  | 0.25 | 0.09 | 0.08 | 0.47 | 0.25 | 0.10 | 0.08 | 0.47 |
|  | **PDW** | Early |  | 11.39 | 0.94 | 9.90 | 12.90 |  | 11.25 | 1.20 | 9.80 | 14.20 | 11.30 | 1.41 | 9.80 | 14.20 |
|  |  | Mid |  | 16.89 | 22.64 | 10.40 | 113.00 |  | 12.07 | 1.00 | 9.70 | 13.10 | 12.04 | 1.00 | 9.70 | 13.10 |
|  |  | Late |  | 11.80 | 1.14 | 9.70 | 14.30 |  | 11.55 | 0.85 | 10.30 | 13.00 | 11.32 | 1.01 | 10.30 | 13.00 |
|  |  | Dry |  | 12.07 | 0.86 | 10.70 | 13.50 |  | 11.73 | 0.93 | 11.10 | 12.80 | - | - | - | - |
|  |  | Total |  | 13.39 | 13.00 | 9.70 | 113.00 |  | 11.66 | 1.05 | 9.70 | 14.20 | 11.63 | 1.17 | 9.70 | 14.20 |
| **Biochemistry** | **Total Protein** | Early |  | 8.64 | 0.95 | 6.70 | 10.20 |  | 8.11 | 0.98 | 6.30 | 9.90 | 8.06 | 0.98 | 6.30 | 9.30 |
|  |  | Mid |  | 8.64 | 1.09 | 6.40 | 11.00 |  | 8.08 | 0.97 | 5.60 | 9.30 | 8.06 | 1.03 | 5.60 | 9.10 |
|  |  | Late |  | 9.29 | 1.13 | 7.40 | 11.50 |  | 8.92 | 0.87 | 7.60 | 10.50 | 9.07 | 1.01 | 7.60 | 10.50 |
|  |  | Dry |  | 8.17 | 1.16 | 6.30 | 9.60 |  | 8.60 | 1.77 | 7.00 | 10.50 | - | - | - | - |
|  |  | Total |  | 8.73 | 1.11 | 6.30 | 11.50 |  | 8.34 | 1.04 | 5.60 | 10.50 | 8.28 | 1.06 | 5.60 | 10.50 |
|  | **Albumin** | Early |  | 2.89 | 0.47 | 2.20 | 3.90 |  | 3.11 | 0.54 | 2.10 | 3.70 | 3.00 | 0.57 | 2.10 | 3.70 |
|  |  | Mid |  | 2.81 | 0.33 | 2.30 | 3.70 |  | 2.95 | 0.51 | 2.00 | 4.00 | 2.83 | 0.45 | 2.00 | 3.60 |
|  |  | Late |  | 2.75 | 0.48 | 2.00 | 3.80 |  | 3.27 | 0.74 | 2.40 | 4.70 | 3.02 | 0.62 | 2.50 | 3.90 |
|  |  | Dry |  | 3.73 | 0.26 | 3.20 | 4.20 |  | 3.53 | 0.55 | 2.90 | 3.90 | - | - | - | - |
|  |  | Total |  | 2.96 | 0.52 | 2.00 | 4.20 |  | 3.12 | 0.60 | 2.00 | 4.70 | 2.93 | 0.52 | 2.00 | 3.90 |
|  | **Globulin** | Early |  | 5.75 | 1.15 | 3.80 | 8.00 |  | 5.00 | 1.02 | 4.00 | 7.20 | 5.06 | 0.81 | 4.20 | 6.80 |
|  |  | Mid |  | 5.83 | 1.26 | 3.60 | 8.70 |  | 5.12 | 0.90 | 3.60 | 6.30 | 5.23 | 0.87 | 3.60 | 6.30 |
|  |  | Late |  | 6.54 | 1.27 | 4.60 | 8.90 |  | 5.65 | 1.15 | 4.40 | 7.70 | 6.05 | 0.95 | 4.60 | 6.80 |
|  |  | Dry |  | 4.43 | 1.05 | 2.90 | 5.70 |  | 5.07 | 2.27 | 3.20 | 7.60 | - | - | - | - |
|  |  | Total |  | 5.78 | 1.34 | 2.90 | 8.90 |  | 5.22 | 1.10 | 3.20 | 7.70 | 5.36 | 0.92 | 3.60 | 6.80 |
|  | **A/G ratio** | Early |  | 0.53 | 0.18 | 0.28 | 1.00 |  | 0.65 | 0.18 | 0.37 | 0.88 | 0.60 | 0.14 | 0.37 | 0.82 |
|  |  | Mid |  | 0.51 | 0.16 | 0.26 | 0.95 |  | 0.60 | 0.16 | 0.41 | 0.95 | 0.55 | 0.12 | 0.41 | 0.78 |
|  |  | Late |  | 0.44 | 0.16 | 0.29 | 0.80 |  | 0.62 | 0.23 | 0.31 | 1.04 | 0.51 | 0.16 | 0.39 | 0.80 |
|  |  | Dry |  | 0.89 | 0.23 | 0.68 | 1.28 |  | 0.82 | 0.41 | 0.38 | 1.19 | - | - | - | - |
|  |  | Total |  | 0.56 | 0.22 | 0.26 | 1.28 |  | 0.63 | 0.21 | 0.31 | 1.19 | 0.56 | 0.13 | 0.37 | 0.82 |
|  | **Total Bilirubin** | Early |  | 0.55 | 0.39 | 0.09 | 1.63 |  | 0.64 | 0.75 | 0.22 | 3.01 | 0.72 | 0.89 | 0.23 | 3.01 |
|  |  | Mid |  | 0.62 | 0.40 | 0.22 | 1.57 |  | 0.43 | 0.21 | 0.13 | 0.98 | 0.47 | 0.23 | 0.13 | 0.98 |
|  |  | Late |  | 0.59 | 0.35 | 0.23 | 1.49 |  | 0.93 | 1.41 | 0.25 | 5.26 | 1.23 | 1.98 | 0.35 | 5.26 |
|  |  | Dry |  | 0.41 | 0.24 | 0.19 | 0.96 |  | 0.39 | 0.19 | 0.21 | 0.59 | - | - | - | - |
|  |  | Total |  | 0.56 | 0.36 | 0.09 | 1.63 |  | 0.62 | 0.84 | 0.13 | 5.26 | 0.72 | 1.05 | 0.13 | 5.26 |
|  | **Ca** | Early |  | 9.47 | 0.62 | 8.00 | 10.40 |  | 9.37 | 0.78 | 7.40 | 10.40 | 9.21 | 0.86 | 7.40 | 10.00 |
|  |  | Mid |  | 9.51 | 0.84 | 7.00 | 10.50 |  | 9.45 | 0.83 | 7.10 | 10.80 | 9.42 | 0.94 | 7.10 | 10.80 |
|  |  | Late |  | 9.45 | 0.72 | 8.00 | 10.20 |  | 9.78 | 0.47 | 9.00 | 10.40 | 9.63 | 0.38 | 9.10 | 9.90 |
|  |  | Dry |  | 9.99 | 0.70 | 8.50 | 11.10 |  | 9.77 | 0.40 | 9.30 | 10.00 | - | - | - | - |
|  |  | Total |  | 9.56 | 0.74 | 7.00 | 11.10 |  | 9.54 | 0.72 | 7.10 | 10.80 | 9.40 | 0.81 | 7.10 | 10.80 |
|  | **P** | Early |  | 6.10 | 0.91 | 5.10 | 8.30 |  | 5.74 | 1.10 | 4.00 | 8.30 | 5.68 | 1.24 | 4.00 | 8.30 |
|  |  | Mid |  | 6.12 | 1.31 | 3.90 | 8.10 |  | 5.99 | 0.97 | 4.40 | 7.50 | 5.98 | 1.07 | 4.40 | 7.50 |
|  |  | Late |  | 6.77 | 0.79 | 5.20 | 7.90 |  | 7.00 | 1.36 | 5.50 | 10.40 | 7.10 | 1.71 | 5.80 | 10.40 |
|  |  | Dry |  | 7.16 | 1.69 | 4.40 | 9.70 |  | 5.90 | 1.23 | 5.00 | 7.30 | - | - | - | - |
|  |  | Total |  | 6.43 | 1.21 | 3.90 | 9.70 |  | 6.18 | 1.21 | 4.00 | 10.40 | 6.13 | 1.35 | 4.00 | 10.40 |
|  | **Mg** | Early |  | 2.14 | 0.31 | 1.42 | 2.65 |  | 2.19 | 0.35 | 1.54 | 2.64 | 2.20 | 0.37 | 1.54 | 2.64 |
|  |  | Mid |  | 2.15 | 0.38 | 1.41 | 2.83 |  | 2.04 | 0.35 | 1.22 | 2.64 | 2.09 | 0.38 | 1.22 | 2.64 |
|  |  | Late |  | 2.11 | 0.33 | 1.38 | 2.72 |  | 2.22 | 0.36 | 1.64 | 2.93 | 2.29 | 0.29 | 1.91 | 2.65 |
|  |  | Dry |  | 2.14 | 0.44 | 1.78 | 3.10 |  | 2.31 | 0.25 | 2.02 | 2.48 | - | - | - | - |
|  |  | Total |  | 2.14 | 0.35 | 1.38 | 3.10 |  | 2.15 | 0.35 | 1.22 | 2.93 | 2.17 | 0.36 | 1.22 | 2.65 |
|  | **NEFA** | Early |  | 0.51 | 0.33 | 0.06 | 1.05 |  | 0.48 | 0.21 | 0.13 | 0.76 | 0.45 | 0.22 | 0.13 | 0.76 |
|  |  | Mid |  | 0.28 | 0.14 | 0.09 | 0.60 |  | 0.25 | 0.16 | 0.12 | 0.71 | 0.25 | 0.17 | 0.12 | 0.71 |
|  |  | Late |  | 0.23 | 0.19 | 0.10 | 0.84 |  | 0.32 | 0.19 | 0.16 | 0.71 | 0.30 | 0.21 | 0.16 | 0.71 |
|  |  | Dry |  | 0.24 | 0.07 | 0.12 | 0.35 |  | 0.26 | 0.14 | 0.09 | 0.34 | - | - | - | - |
|  |  | Total |  | 0.32 | 0.24 | 0.06 | 1.05 |  | 0.34 | 0.20 | 0.09 | 0.76 | 0.33 | 0.21 | 0.12 | 0.76 |
|  | **BOHB** | Early |  | 0.71 | 0.31 | 0.33 | 1.54 |  | 0.67 | 0.36 | 0.32 | 1.48 | 0.78 | 0.38 | 0.32 | 1.48 |
|  |  | Mid |  | 0.67 | 0.23 | 0.29 | 1.20 |  | 0.62 | 0.23 | 0.34 | 1.06 | 0.61 | 0.22 | 0.34 | 1.03 |
|  |  | Late |  | 0.58 | 0.17 | 0.35 | 0.97 |  | 0.54 | 0.27 | 0.05 | 1.12 | 0.54 | 0.35 | 0.05 | 1.12 |
|  |  | Dry |  | 0.49 | 0.09 | 0.35 | 0.63 |  | 0.50 | 0.12 | 0.36 | 0.58 | - | - | - | - |
|  |  | Total |  | 0.64 | 0.24 | 0.29 | 1.54 |  | 0.60 | 0.28 | 0.05 | 1.48 | 0.65 | 0.31 | 0.05 | 1.48 |
|  | **Lactate** | Early |  | 2.60 | 0.68 | 1.69 | 3.87 |  | 2.63 | 0.90 | 1.10 | 3.62 | 2.58 | 0.97 | 1.10 | 3.62 |
|  |  | Mid |  | 2.84 | 1.08 | 1.47 | 5.23 |  | 2.77 | 0.82 | 1.34 | 4.84 | 2.74 | 0.92 | 1.34 | 4.84 |
|  |  | Late |  | 2.62 | 0.66 | 1.64 | 3.93 |  | 3.02 | 1.21 | 1.23 | 5.39 | 3.17 | 1.35 | 1.60 | 5.39 |
|  |  | Dry |  | 3.50 | 1.09 | 2.24 | 5.22 |  | 3.07 | 0.75 | 2.25 | 3.73 | - | - | - | - |
|  |  | Total |  | 2.82 | 0.92 | 1.47 | 5.23 |  | 2.81 | 0.94 | 1.10 | 5.39 | 2.78 | 1.02 | 1.10 | 5.39 |
| **Enzymes** | **AST** | Early |  | 72.75 | 29.04 | 46.00 | 171.00 |  | 78.46 | 24.09 | 48.00 | 131.00 | 75.33 | 21.26 | 48.00 | 110.00 |
|  |  | Mid |  | 76.15 | 26.49 | 43.00 | 144.00 |  | 73.71 | 24.16 | 49.00 | 159.00 | 76.00 | 28.60 | 49.00 | 159.00 |
|  |  | Late |  | 66.53 | 19.37 | 45.00 | 104.00 |  | 70.83 | 12.72 | 53.00 | 95.00 | 73.67 | 16.17 | 53.00 | 95.00 |
|  |  | Dry |  | 86.56 | 62.58 | 56.00 | 252.00 |  | 70.00 | 25.87 | 47.00 | 98.00 | - | - | - | - |
|  |  | Total |  | 74.40 | 33.17 | 43.00 | 252.00 |  | 74.07 | 21.24 | 47.00 | 159.00 | 75.26 | 23.16 | 48.00 | 159.00 |
|  | **CK** | Early |  | 123.56 | 67.70 | 57.00 | 285.00 |  | 184.77 | 228.12 | 37.00 | 782.00 | 229.33 | 264.54 | 39.00 | 782.00 |
|  |  | Mid |  | 165.35 | 235.34 | 54.00 | 1137.00 |  | 102.29 | 32.50 | 57.00 | 183.00 | 108.83 | 35.11 | 65.00 | 183.00 |
|  |  | Late |  | 114.53 | 72.62 | 28.00 | 318.00 |  | 113.33 | 51.27 | 61.00 | 204.00 | 93.50 | 32.95 | 61.00 | 139.00 |
|  |  | Dry |  | 1245.89 | 3274.62 | 62.00 | 9971.00 |  | 772.67 | 1220.57 | 57.00 | 2182.00 | - | - | - | - |
|  |  | Total |  | 303.58 | 1278.29 | 28.00 | 9971.00 |  | 173.76 | 332.28 | 37.00 | 2182.00 | 145.59 | 161.06 | 39.00 | 782.00 |
|  | **LDH** | Early |  | 931.06 | 372.51 | 453.00 | 2173.00 |  | 889.85 | 154.22 | 603.00 | 1094.00 | 886.67 | 178.60 | 603.00 | 1094.00 |
|  |  | Mid |  | 881.20 | 217.56 | 504.00 | 1362.00 |  | 914.00 | 356.91 | 464.00 | 2113.00 | 949.92 | 393.06 | 601.00 | 2113.00 |
|  |  | Late |  | 800.40 | 210.15 | 434.00 | 1185.00 |  | 791.58 | 141.84 | 535.00 | 1090.00 | 840.17 | 183.99 | 535.00 | 1090.00 |
|  |  | Dry |  | 855.33 | 190.29 | 657.00 | 1305.00 |  | 795.00 | 275.09 | 524.00 | 1074.00 | - | - | - | - |
|  |  | Total |  | 870.42 | 261.21 | 434.00 | 2173.00 |  | 866.44 | 253.36 | 464.00 | 2113.00 | 904.44 | 289.32 | 535.00 | 2113.00 |
|  | **GLDH** | Early |  | 22.47 | 18.50 | 6.40 | 75.40 |  | 35.59 | 38.07 | 0.00 | 121.00 | 31.59 | 41.32 | 0.00 | 121.00 |
|  |  | Mid |  | 31.86 | 35.21 | 7.20 | 163.90 |  | 40.10 | 34.42 | 9.40 | 137.70 | 47.16 | 38.39 | 13.10 | 137.70 |
|  |  | Late |  | 32.55 | 26.76 | 12.80 | 105.60 |  | 20.10 | 12.78 | 8.00 | 55.30 | 23.38 | 16.74 | 10.10 | 55.30 |
|  |  | Dry |  | 57.72 | 116.37 | 9.00 | 367.00 |  | 10.43 | 3.02 | 8.40 | 13.90 | - | - | - | - |
|  |  | Total |  | 33.42 | 51.46 | 6.40 | 367.00 |  | 31.49 | 31.09 | 0.00 | 137.70 | 36.69 | 36.10 | 0.00 | 137.70 |
|  | **Cortisol** | Early |  | 7.17 | 9.39 | 1.02 | 35.48 |  | 10.94 | 14.76 | 0.56 | 43.86 | 11.50 | 17.56 | 0.56 | 43.86 |
|  |  | Mid |  | 4.26 | 2.72 | 0.77 | 10.08 |  | 6.81 | 6.02 | 1.25 | 19.36 | 6.34 | 5.66 | 1.25 | 19.36 |
|  |  | Late |  | 7.10 | 5.84 | 1.89 | 20.14 |  | 9.66 | 5.61 | 2.90 | 19.12 | 8.07 | 6.61 | 2.90 | 19.12 |
|  |  | Dry |  | 10.75 | 5.37 | 5.55 | 18.39 |  | 11.45 | 9.47 | 1.28 | 20.01 | - | - | - | - |
|  |  | Total |  | 6.81 | 6.41 | 0.77 | 35.48 |  | 9.11 | 9.51 | 0.56 | 43.86 | 8.54 | 11.36 | 0.56 | 43.86 |

Supplementary Tab. 2. Descriptive statistics (mean, standard deviation, minimum and maximum) of hematological, biochemical and enzyme activities analyses, and cortisol determination sorted by the lactation phase and the serological and clinical status of cows in a dairy cattle herd endemically infected by bovine besnoitiosis. The lactation phase was classified as follows: Early = 0-120 d, Mid = 121-250 d, Late= 251-305 d, and Dry. Serological status (seronegative or seropositive) was determined according to Western Blot results while as clinically affected cows are meant those animals with the presence of clinical signs of the disease. Blank spaces (-) are due to the lack of clinically affected cows in the dry phase.
