# Supplementary material for: Exploring alterations in hematological and biochemical parameters, enzyme activities and serum cortisol in Besnoitia besnoiti naturally infected dairy cattle
Source: Parasit Vectors. 2021 Mar 15;14:154. doi: 10.1186/s13071-021-04626-4 (PMC7962361; doi:10.1186/s13071-021-04626-4)
Supplement: Supplementary file 3 — Additional file 3: Table S3. Results of the significative variables in the GLM analysis regarding the variation on laboratory parameters according to number of parturitions and phase of lactation. Descriptive statistics (mean, standard deviation, minimum and maximum) of the considered parameters were also included. [file 13071_2021_4626_MOESM3_ESM.docx]

| **Response Variable** | **Predictor** | **Category** | **Mean (SD)** | **Min-Max** | **β^a^** | **SE^b^** | **Wald**  **Chi-Square** | **OR (95% CI)** | **P-value** | **AIC** |
| --- | --- | --- | --- | --- | --- | --- | --- | --- | --- | --- |
| **RBC** | Number of parturitions |  |  |  |  |  | 11.038 |  | 0.001 | 183.072 |
|  |  | Primiparous | 6.36 (0.56) | 5.18-7.55 | 0.378 | 0.1137 |  | 1.459 (1.168-1.823) | 0.001 |  |
|  |  | Multiparous | 5.86 (0.66) | 4.67-7.23 | 0 |  |  | 1 |  |  |
|  | Phase of lactation |  |  |  |  |  | 21.433 |  | 0.0001 |  |
|  |  | Early | 5.65 (0.67) ^a^ | 4.67-7.30 | -0.292 | 0.2204 |  | 0.746 (0.485-1.150) | 0.185 |  |
|  |  | Mid | 6.36 (0.57) ^b^ | 5.10-7.55 | 0.307 | 0.2179 |  | 1.359 (0.887-2.083) | 0.159 |  |
|  |  | Late | 6.24 (0.47) ^b^ | 5.32-7.14 | 0.245 | 0.2236 |  | 1.277 (0.824-1.980) | 0.274 |  |
|  |  | Dry | 6.37 (0.97) ^ab^ | 4.93-8.53 | 0 |  |  | 1 |  |  |
| **Hb** | Phase of lactation |  |  |  |  |  | 8.794 |  | 0.032 | 397.236 |
|  |  | Early | 11.64 (2.05) ^a^ | 3.81-14.34 | -0.685 | 0.6287 |  | 0.504 (0.147-1.729) | 0.276 |  |
|  |  | Mid | 12.67 (1.41) ^b^ | 9.71-15.88 | 0.350 | 0.6182 |  | 1.419 (0.423-4.767) | 0.571 |  |
|  |  | Late | 12.66 (1.28) ^b^ | 9.67-15.59 | 0.336 | 0.6382 |  | 1.399 (0.401-4.888) | 0.599 |  |
|  |  | Dry | 12.40 (1.36) ^ab^ | 10.40-14.89 | 0 |  |  | 1 |  |  |
| **Ht** | Phase of lactation |  |  |  |  |  | 12.343 |  | 0.006 | 486.657 |
|  |  | Early | 25.68 (2.70) | 20.70-31.20 | -2.419 | 0.9704 |  | 0.089 (0.013-0.596) | 0.013 |  |
|  |  | Mid | 27.35 (2.28) ^a^ | 21.50-31.10 | -0.751 | 0.9542 |  | 0.472 (0.073-3.061) | 0.431 |  |
|  |  | Late | 27.48 (2.37) ^a^ | 23.60-32.20 | -0.622 | 0.9851 |  | 0.537 (0.078-3.701) | 0.528 |  |
|  |  | Dry | 28.42 (3.14) ^a^ | 23.90-32.40 | 0 |  |  | 1 |  |  |
| **MCV** | Number of parturitions |  |  |  |  |  | 5.587 |  | 0.018 | 526.958 |
|  |  | Primiparous | 43.41 (3.04) | 37-49 | -1.427 | 0.6035 |  | 0.240 (0.074-0.784) | 0.018 |  |
|  |  | Multiparous | 45.30 (3.25) | 38-56 | 0 |  |  | 1 |  |  |
|  | Phase of lactation |  |  |  |  |  | 12.943 |  | 0.005 |  |
|  |  | Early | 45.55 (2.83) ^ac^ | 39-50 | -1.572 | 1.1699 |  | 0.208 (0.021-2.055) | 0.179 |  |
|  |  | Mid | 43.14 (3.28) ^b^ | 38-56 | -3.552 | 1.1567 |  | 0.29 (0.003-0.277) | 0.002 |  |
|  |  | Late | 44.26 (3.28) ^ab^ | 37-49 | -2.642 | 1.1871 |  | 0.071 (0.007-0.730) | 0.026 |  |
|  |  | Dry | 45.17 (6.09) ^c^ | 32-50 | 0 |  |  | 1 |  |  |
| **RDW** | Phase of lactation |  |  |  |  |  | 18.852 |  | 0.000 | 274.083 |
|  |  | Early | 14.76 (0.86) ^b^ | 13.40-17.30 | -0.200 | 0.2826 |  | 0.819 (0.470-1.424) | 0.479 |  |
|  |  | Mid | 15.57 (0.79) ^a^ | 13.70-17.20 | 0.617 | 0.2761 |  | 1.854 (1.079-3.185) | 0.025 |  |
|  |  | Late | 15.40 (0.76) ^ac^ | 14.00-16.70 | 0.445 | 0.2884 |  | 1.561 (0.887-2.747) | 0.122 |  |
|  |  | Dry | 14.96 (1.12) ^bc^ | 13.60-17.50 | 0 |  |  | 1 |  |  |
| **Ca** | Number of parturitions |  |  |  |  |  | 7.995 |  | 0.005 | 217.610 |
|  |  | Primiparous | 9.73 (0.69) | 7.1-10.8 | 0.390 | 0.1381 |  | 1.478 (1.127-1.937) | 0.005 |  |
|  |  | Multiparous | 9.34 (0.70) | 7.0-10.4 | 0 |  |  | 1 |  |  |
| **P** | Phase of lactation |  |  |  |  |  | 12.834 |  | 0.005 | 311.434 |
|  |  | Early | 5.94 (0.99) ^a^ | 4.0-8.3 | -0.162 | 0.4297 |  | 0.850 (0.366-1.974) | 0.706 |  |
|  |  | Mid | 6.06 (1.15) ^a^ | 3.9-8.1 | -0.041 | 0.4196 |  | 0.960 (0.422-2.185) | 0.923 |  |
|  |  | Late | 6.87 (1.06) ^b^ | 5.2-10.4 | 0.774 | 0.4332 |  | 2.169 (0.928-5.069) | 0.074 |  |
|  |  | Dry | 6.84 (1.64) ^ab^ | 4.4-9.7 | 0 |  |  | 1 |  |  |
| **NEFA** | Phase of lactation |  |  |  |  |  | 27.916 |  | 0.000 | 30.288 |
|  |  | Early | 0.49 (0.28) | 0.06-1.05 | 0.268 | 0.0790 |  | 1.307 (1.120-1.526) | 0.001 |  |
|  |  | Mid | 0.27 (0.15) ^a^ | 0.09-0.71 | 0.041 | 0.0771 |  | 1.042 (0.896-1.212) | 0.596 |  |
|  |  | Late | 0.27 (0.19) ^a^ | 0.10-0.84 | 0.048 | 0.0800 |  | 1.049 (0.897-1.227) | 0.548 |  |
|  |  | Dry | 0.24 (0.09) ^a^ | 0.09-0.35 | 0 |  |  | 1 |  |  |
| **AST** | Number of parturitions |  |  |  |  |  | 8.204 |  | 0.004 | 38.728 |
|  |  | Primiparous | 78.42 (36.01) | 43-252 | 0.679 | 0.1958 |  | 1.971 (1.343-2.893) | 0.001 |  |
|  |  | Multiparous | 70.73 (21.50) | 44-171 | 0 |  |  | 1 |  |  |
|  | Phase of lactation |  |  |  |  |  | 5.083 |  | 0.166 |  |
|  |  | Early | 75.31 (26.62) ^ab^ | 46-171 | 0.169 | 0.1334 |  | 1.184 (0.911-1.538) | 0.206 |  |
|  |  | Mid | 75.03 (25.12) ^ab^ | 43-159 | 0.110 | 0.1385 |  | 1.116 (0.851-1.464) | 0.427 |  |
|  |  | Late | 68.44 (16.59) ^a^ | 45-104 | 0.062 | 0.1385 |  | 1.064 (0.811-1.396) | 0.654 |  |
|  |  | Dry | 82.42 (55.01) ^b^ | 47-252 | 0 |  |  | 1 |  |  |
|  | Number of parturitions  X  Phase of lactation |  |  |  |  |  | 10.750 |  | 0.013 |  |
|  |  | Primiparous  Early | 71.75 (15.15) | 51-94 | -0.728 | 0.2253 |  | 0.483 (0.311-0.751) | 0.001 |  |
|  |  | Primiparous  Mid | 77.91 (30.21) | 43-159 | -0.608 | 0.2154 |  | 0.545 (0.357-0.831) | 0.005 |  |
|  |  | Primiparous  Late | 71.50 (20.68) | 45-104 | -0.624 | 0.2216 |  | 0.536 (0.347-0.828) | 0.005 |  |
|  |  | Primiparous  Dry | 127.67 (108.09) | 56-252 | 0 |  |  | 1 |  |  |
|  |  | Multiparous  Early | 76.67 (30.08) | 46-171 | 0 |  |  | 1 |  |  |
|  |  | Multiparous  Mid | 70.80 (14.89) | 44-108 | 0 |  |  | 1 |  |  |
|  |  | Multiparous  Late | 66.00 (12.68) | 48-95 | 0 |  |  | 1 |  |  |
|  |  | Multiparous  Dry | 59.80 (8.81) | 47-70 | 0 |  |  | 1 |  |  |
| **CK** | Number of parturitions |  |  |  |  |  | 16.221 |  | 0.000 | 740.220 |
|  |  | Primiparous | 339.04 (1469.46) | 28-9971 | 32.329 | 5.8866 |  | 1.098E+14 (1070923246-1.125E+19) | 0.000 |  |
|  |  | Multiparous | 147.21 (188.48) | 37-1137 | 0 |  |  | 1 |  |  |
|  | Phase of lactation |  |  |  |  |  | 28.171 |  | 0.000 |  |
|  |  | Early | 151.00 (160.37) ^a^ | 37-782 | -0.102 | 3.9935 |  | 0.903 (0.000-2265.046) | 0.980 |  |
|  |  | Mid | 136.38 (175.26) ^a^ | 54-1137 | 0.118 | 4.1625 |  | 1.125 (0.000-3929.539) | 0.977 |  |
|  |  | Late | 114.00 (62.87) ^a^ | 28-318 | -0.644 | 4.1625 |  | 0.525 (0.000-1834.041) | 0.877 |  |
|  |  | Dry | 1127.58 (2848.74) | 57-9971 | 0 |  |  | 1 |  |  |
|  | Number of parturitions  X  Phase of lactation |  |  |  |  |  | 28.298 |  | 0.000 |  |
|  |  | Primiparous  Early | 128.75 (64.88) | 62-256 | -32.707 | 6.6951 |  | 6.244E-15 (1.249E-20-3.121E-9) | 0.000 |  |
|  |  | Primiparous  Mid | 110.59 (38.69) | 54-230 | -32.965 | 6.4759 |  | 4.823E-15 (1.483E-20-1.569E-9) | 0.000 |  |
|  |  | Primiparous  Late | 134.00 (73.14) | 28-318 | -31.969 | 6.6632 |  | 1.306E-14 (2.781E-20-6.132E-9) | 0.000 |  |
|  |  | Primiparous  Dry | 3395.33 (5694.78) | 77-9971 | 0 |  |  | 1 |  |  |
|  |  | Multiparous  Early | 159.48 (185.09) | 37-782 | 0 |  |  | 1 |  |  |
|  |  | Multiparous  Mid | 174.20 (272.32) | 57-1137 | 0 |  |  | 1 |  |  |
|  |  | Multiparous  Late | 98.00 (50.20) | 57-204 | 0 |  |  | 1 |  |  |
|  |  | Multiparous  Dry | 162.40 (189.53) | 57-499 | 0 |  |  | 1 |  |  |
| **LDH** | Number of parturitions |  |  |  |  |  | 5.144 |  | 0.023 | 480.299 |
|  |  | Primiparous | 930.51 (268.04) | 434-2113 | 1.150 | 0.5069 |  | 3.157 (1.169-8.528) | 0.023 |  |
|  |  | Multiparous | 815.54 (245.50) | 453-2173 | 0 |  |  | 1 |  |  |

Supplementary Table 3. Results of the significative variables to the GLM analysis regarding the variation on laboratory parameters according to number of parturitions and phase of lactation. Descriptive statistics (mean, standard deviation, minimum and maximum) of the considered parameters were also included. Mean values of each parameter per each phase of lactation with different superscript letters are statistically different from each other (p−value <0.05, GLM, pairwise comparison), while those with the same superscript letters are not statistically different from each other (p−value >0.05, GLM, pairwise comparison). The number of parturitions was classified as follows: Primiparous = one parturition, Multiparous = two or more parturitions. The phase of lactation was classified as follows: Early = 0-120 days, Mid = 121-250 days, Late= 251-305 days, and Dry.
