# Supplementary material for: Exploring alterations in hematological and biochemical parameters, enzyme activities and serum cortisol in Besnoitia besnoiti naturally infected dairy cattle
Source: Parasit Vectors. 2021 Mar 15;14:154. doi: 10.1186/s13071-021-04626-4 (PMC7962361; doi:10.1186/s13071-021-04626-4)
Supplement: Supplementary file 4 — Additional file 4: Tables S4. Selection of final model by backward elimination according to the best Akaike's information criterion (AIC). AIC values were provided for the full models and for each step corresponding to the removal of the least statistically significant variable, until the final model was obtained. [file 13071_2021_4626_MOESM4_ESM.docx]

**Supplementary Tables 4**

| **Table 4.1 –** Selection of final model by backward elimination according to the best Akaike's information criterion (AIC). The full model, having Lymphocytes (continuous variable) as response variable, contained the following independent variables: the seropositivity to *B. besnoiti* (dichotomous variable: presence vs. absence of antibodies based on Western Blot results), the number of parturitions (dichotomous variable: primiparous vs. multiparous cows), the phase of lactation (ordinal variable: early, mid, late, and dry), and all their two-way interactions. | | |
| --- | --- | --- |
| **Step of backward elimination** | **Eliminated variable** | **AIC** |
| Full model | -- | 81.652 |
| Step 1 | phase of lactation × seropositivity to *B. besnoiti* | 84.239 |
| Step 2 | number of parturitions × phase of lactation | 81.163 |
| Step 3 | phase of lactation | 80.636 |
| Step 4 | number of parturitions | 80.636 |

| **Table 4.2 –** Selection of final model by backward elimination according to the best Akaike's information criterion (AIC). The full model, having Lymphocytes (continuous variable) as response variable, contained the following independent variables: the clinical signs of bovine besnoitiosis (dichotomous variable: seronegative or asymptomatic seropositive animals vs. clinically affected cows), the number of parturitions (dichotomous variable: primiparous vs. multiparous cows), the phase of lactation (ordinal variable: early, mid, late, and dry), and all their two-way interactions. | | |
| --- | --- | --- |
| **Step of backward elimination** | **Eliminated variable** | **AIC** |
| Full model | -- | 64.263 |
| Step 1 | phase of lactation × clinical signs of bovine besnoitiosis | 65.468 |
| Step 2 | number of parturitions × phase of lactation | 65.155 |
| Step 3 | phase of lactation | 64.738 |
| Step 4 | number of parturitions | 64.738 |

| **Table 4.3 –** Selection of final model by backward elimination according to the best Akaike's information criterion (AIC). The full model, having Granulocytes (continuous variable) as response variable, contained the following independent variables: the seropositivity to *B. besnoiti* (dichotomous variable: presence vs. absence of antibodies based on Western Blot results), the number of parturitions (dichotomous variable: primiparous vs. multiparous cows), the phase of lactation (ordinal variable: early, mid, late, and dry), and all their two-way interactions. | | |
| --- | --- | --- |
| **Step of backward elimination** | **Eliminated variable** | **AIC** |
| Full model | -- | 81.652 |
| Step 1 | phase of lactation × seropositivity to *B. besnoiti* | 84.239 |
| Step 2 | number of parturitions × phase of lactation | 81.163 |
| Step 3 | phase of lactation | 80.636 |
| Step 4 | number of parturitions | 80.636 |

| **Table 4.4 –** Selection of final model by backward elimination according to the best Akaike's information criterion (AIC). The full model, having Granulocytes (continuous variable) as response variable, contained the following independent variables: the clinical signs of bovine besnoitiosis (dichotomous variable: seronegative or asymptomatic seropositive animals vs. clinically affected cows), the number of parturitions (dichotomous variable: primiparous vs. multiparous cows), the phase of lactation (ordinal variable: early, mid, late, and dry), and all their two-way interactions. | | |
| --- | --- | --- |
| **Step of backward elimination** | **Eliminated variable** | **AIC** |
| Full model | -- | 65.263 |
| Step 1 | phase of lactation × clinical signs of bovine besnoitiosis | 65.468 |
| Step 2 | number of parturitions × phase of lactation | 65.155 |
| Step 3 | phase of lactation | 64.738 |
| Step 4 | number of parturitions | 64.738 |

| **Table 4.5 –** Selection of final model by backward elimination according to the best Akaike's information criterion (AIC). The full model, having Total proteins (continuous variable) as response variable, contained the following independent variables: the seropositivity to *B. besnoiti* (dichotomous variable: presence vs. absence of antibodies based on Western Blot results), the number of parturitions (dichotomous variable: primiparous vs. multiparous cows), the phase of lactation (ordinal variable: early, mid, late, and dry), and all their two-way interactions. | | |
| --- | --- | --- |
| **Step of backward elimination** | **Eliminated variable** | **AIC** |
| Full model | -- | 317.150 |
| Step 1 | number of parturitions × seropositivity to *B. besnoiti* | 315.330 |
| Step 2 | number of parturitions × phase of lactation | 310.444 |
| Step 3 | phase of lactation × seropositivity to *B. besnoiti* | 305.722 |
| Step 4 | number of parturitions | 304.313 |

| **Table 4.6 –** Selection of final model by backward elimination according to the best Akaike's information criterion (AIC). The full model, having Albumin (continuous variable) as response variable, contained the following independent variables: the seropositivity to *B. besnoiti* (dichotomous variable: presence vs. absence of antibodies based on Western Blot results), the number of parturitions (dichotomous variable: primiparous vs. multiparous cows), the phase of lactation (ordinal variable: early, mid, late, and dry), and all their two-way interactions. | | |
| --- | --- | --- |
| **Step of backward elimination** | **Eliminated variable** | **AIC** |
| Full model | -- | 163.328 |
| Step 1 | number of parturitions × phase of lactation | 158.458 |
| Step 2 | number of parturitions × seropositivity to *B. besnoiti* | 157.170 |
| Step 3 | number of parturitions | 155.255 |
| Step 4 | phase of lactation × seropositivity to *B. besnoiti* | 153.895 |

| **Table 4.7 –** Selection of final model by backward elimination according to the best Akaike's information criterion (AIC). The full model, having Globulin (continuous variable) as response variable, contained the following independent variables: the seropositivity to *B. besnoiti* (dichotomous variable: presence vs. absence of antibodies based on Western Blot results), the number of parturitions (dichotomous variable: primiparous vs. multiparous cows), the phase of lactation (ordinal variable: early, mid, late, and dry), and all their two-way interactions. | | |
| --- | --- | --- |
| **Step of backward elimination** | **Eliminated variable** | **AIC** |
| Full model | -- | 337.148 |
| Step 1 | number of parturitions × phase of lactation | 333.134 |
| Step 2 | number of parturitions × seropositivity to *B. besnoiti* | 331.533 |
| Step 3 | number of parturitions | 330.043 |
| Step 4 | phase of lactation × seropositivity to *B. besnoiti* | 326.879 |

| **Table 4.8 –** Selection of final model by backward elimination according to the best Akaike's information criterion (AIC). The full model, having A/G ratio (continuous variable) as response variable, contained the following independent variables: the seropositivity to *B. besnoiti* (dichotomous variable: presence vs. absence of antibodies based on Western Blot results), the number of parturitions (dichotomous variable: primiparous vs. multiparous cows), the phase of lactation (ordinal variable: early, mid, late, and dry), and all their two-way interactions. | | |
| --- | --- | --- |
| **Step of backward elimination** | **Eliminated variable** | **AIC** |
| Full model | -- | 42.561 |
| Step 1 | number of parturitions × phase of lactation | 45.650 |
| Step 2 | number of parturitions × seropositivity to *B. besnoiti* | 46.871 |
| Step 3 | number of parturitions | 48.869 |
| Step 4 | phase of lactation × seropositivity to *B. besnoiti* | 42.458 |

| **Table 4.9 –**Selection of final model by backward elimination according to the best Akaike's information criterion (AIC). The full model, having GLDH (continuous variable) as response variable, contained the following independent variables: the clinical signs of bovine besnoitiosis (dichotomous variable: seronegative or asymptomatic seropositive animals vs. clinically affected cows), the number of parturitions (dichotomous variable: primiparous vs. multiparous cows), the phase of lactation (ordinal variable: early, mid, late, and dry), and all their two-way interactions. | | |
| --- | --- | --- |
| **Step of backward elimination** | **Eliminated variable** | **AIC** |
| Full model | -- | 54.556 |
| Step 1 | number of parturitions × clinical signs of bovine besnoitiosis | 52.579 |
| Step 2 | phase of lactation × clinical signs of bovine besnoitiosis | 50.114 |
| Step 3 | number of parturitions × phase of lactation | 46.645 |
| Step 4 | phase of lactation | 33.850 |

| **Table 4.10 –** Selection of final model by backward elimination according to the best Akaike's information criterion (AIC). The full model, having RBC (continuous variable) as response variable, contained the following independent variables: the seropositivity to *B. besnoiti* (dichotomous variable: presence vs. absence of antibodies based on Western Blot results), the number of parturitions (dichotomous variable: primiparous vs. multiparous cows), the phase of lactation (ordinal variable: early, mid, late, and dry), and all their two-way interactions. | | |
| --- | --- | --- |
| **Step of backward elimination** | **Eliminated variable** | **AIC** |
| Full model | -- | 193.403 |
| Step 1 | phase of lactation × seropositivity to *B. besnoiti* | 188.353 |
| Step 2 | number of parturitions × seropositivity to *B. besnoiti* | 186.381 |
| Step 3 | number of parturitions × phase of lactation | 183.090 |
| Step 4 | seropositivity to *B. besnoiti* | 183.072 |

| **Table 4.11 –** Selection of final model by backward elimination according to the best Akaike's information criterion (AIC). The full model, having RBC (continuous variable) as response variable, contained the following independent variables: the clinical signs of bovine besnoitiosis (dichotomous variable: seronegative or asymptomatic seropositive animals vs. clinically affected cows), the number of parturitions (dichotomous variable: primiparous vs. multiparous cows), the phase of lactation (ordinal variable: early, mid, late, and dry), and all their two-way interactions. | | |
| --- | --- | --- |
| **Step of backward elimination** | **Eliminated variable** | **AIC** |
| Full model | -- | 168.996 |
| Step 1 | number of parturitions × clinical signs of bovine besnoitiosis | 167.023 |
| Step 2 | phase of lactation × clinical signs of bovine besnoitiosis | 164.625 |
| Step 3 | clinical signs of bovine besnoitiosis | 162.663 |
| Step 4 | number of parturitions × phase of lactation | 158.816 |

| **Table 4.12 –** Selection of final model by backward elimination according to the best Akaike's information criterion (AIC). The full model, having Hb (continuous variable) as response variable, contained the following independent variables: the seropositivity to *B. besnoiti* (dichotomous variable: presence vs. absence of antibodies based on Western Blot results), the number of parturitions (dichotomous variable: primiparous vs. multiparous cows), the phase of lactation (ordinal variable: early, mid, late, and dry), and all their two-way interactions. | | |
| --- | --- | --- |
| **Step of backward elimination** | **Eliminated variable** | **AIC** |
| Full model | -- | 409.750 |
| Step 1 | number of parturitions × seropositivity to *B. besnoiti* | 407.839 |
| Step 2 | number of parturitions × phase of lactation | 402.343 |
| Step 3 | phase of lactation × seropositivity to *B. besnoiti* | 398.822 |
| Step 4 | seropositivity to *B. besnoiti* | 397.520 |
| Step 5 | number of parturitions | 397.236 |

| **Table 4.13 –** Selection of final model by backward elimination according to the best Akaike's information criterion (AIC). The full model, having Hb (continuous variable) as response variable, contained the following independent variables: the clinical signs of bovine besnoitiosis (dichotomous variable: seronegative or asymptomatic seropositive animals vs. clinically affected cows), the number of parturitions (dichotomous variable: primiparous vs. multiparous cows), the phase of lactation (ordinal variable: early, mid, late, and dry), and all their two-way interactions. | | |
| --- | --- | --- |
| **Step of backward elimination** | **Eliminated variable** | **AIC** |
| Full model | -- | 462.011 |
| Step 1 | number of parturitions × clinical signs of bovine besnoitiosis | 460.017 |
| Step 2 | number of parturitions × phase of lactation | 455.179 |
| Step 3 | phase of lactation × clinical signs of bovine besnoitiosis | 454.462 |
| Step 4 | number of parturitions | 409.368 |
| Step 5 | clinical signs of bovine besnoitiosis | 397.236 |

| **Table 4.14 –** Selection of final model by backward elimination according to the best Akaike's information criterion (AIC). The full model, having Ht (continuous variable) as response variable, contained the following independent variables: the seropositivity to *B. besnoiti* (dichotomous variable: presence vs. absence of antibodies based on Western Blot results), the number of parturitions (dichotomous variable: primiparous vs. multiparous cows), the phase of lactation (ordinal variable: early, mid, late, and dry), and all their two-way interactions. | | |
| --- | --- | --- |
| **Step of backward elimination** | **Eliminated variable** | **AIC** |
| Full model | -- | 494.289 |
| Step 1 | number of parturitions × seropositivity to *B. besnoiti* | 492.319 |
| Step 2 | number of parturitions × phase of lactation | 488.037 |
| Step 3 | phase of lactation × seropositivity to *B. besnoiti* | 485.018 |
| Step 4 | seropositivity to *B. besnoiti* | 485.680 |
| Step 5 | number of parturitions | 485.657 |

| **Table 4.15 –** Selection of final model by backward elimination according to the best Akaike's information criterion (AIC). The full model, having Ht (continuous variable) as response variable, contained the following independent variables: the clinical signs of bovine besnoitiosis (dichotomous variable: seronegative or asymptomatic seropositive animals vs. clinically affected cows), the number of parturitions (dichotomous variable: primiparous vs. multiparous cows), the phase of lactation (ordinal variable: early, mid, late, and dry), and all their two-way interactions. | | |
| --- | --- | --- |
| **Step of backward elimination** | **Eliminated variable** | **AIC** |
| Full model | -- | 532.735 |
| Step 1 | phase of lactation × clinical signs of bovine besnoitiosis | 528.935 |
| Step 2 | number of parturitions × phase of lactation | 523.827 |
| Step 3 | number of parturitions × clinical signs of bovine besnoitiosis | 522.137 |
| Step 4 | clinical signs of bovine besnoitiosis | 520.138 |
| Step 5 | number of parturitions | 486.657 |

| **Table 4.16 –** Selection of final model by backward elimination according to the best Akaike's information criterion (AIC). The full model, having MCV (continuous variable) as response variable, contained the following independent variables: the seropositivity to *B. besnoiti* (dichotomous variable: presence vs. absence of antibodies based on Western Blot results), the number of parturitions (dichotomous variable: primiparous vs. multiparous cows), the phase of lactation (ordinal variable: early, mid, late, and dry), and all their two-way interactions. | | |
| --- | --- | --- |
| **Step of backward elimination** | **Eliminated variable** | **AIC** |
| Full model | -- | 533.909 |
| Step 1 | number of parturitions × phase of lactation | 529.707 |
| Step 2 | number of parturitions × seropositivity to *B. besnoiti* | 528.559 |
| Step 3 | phase of lactation × seropositivity to *B. besnoiti* | 528.888 |
| Step 4 | seropositivity to *B. besnoiti* | 526.958 |

| **Table 4.17 –** Selection of final model by backward elimination according to the best Akaike's information criterion (AIC). The full model, having MCV (continuous variable) as response variable, contained the following independent variables: the clinical signs of bovine besnoitiosis (dichotomous variable: seronegative or asymptomatic seropositive animals vs. clinically affected cows), the number of parturitions (dichotomous variable: primiparous vs. multiparous cows), the phase of lactation (ordinal variable: early, mid, late, and dry), and all their two-way interactions. | | |
| --- | --- | --- |
| **Step of backward elimination** | **Eliminated variable** | **AIC** |
| Full model | -- | 571.216 |
| Step 1 | number of parturitions × clinical signs of bovine besnoitiosis | 570.161 |
| Step 2 | number of parturitions × phase of lactation | 566.450 |
| Step 3 | phase of lactation × clinical signs of bovine besnoitiosis | 566.306 |
| Step 4 | clinical signs of bovine besnoitiosis | 526.958 |

| **Table 4.18 –** Selection of final model by backward elimination according to the best Akaike's information criterion (AIC). The full model, having RDW (continuous variable) as response variable, contained the following independent variables: the seropositivity to *B. besnoiti* (dichotomous variable: presence vs. absence of antibodies based on Western Blot results), the number of parturitions (dichotomous variable: primiparous vs. multiparous cows), the phase of lactation (ordinal variable: early, mid, late, and dry), and all their two-way interactions. | | |
| --- | --- | --- |
| **Step of backward elimination** | **Eliminated variable** | **AIC** |
| Full model | -- | 279.805 |
| Step 1 | number of parturitions × seropositivity to *B. besnoiti* | 278.010 |
| Step 2 | number of parturitions × phase of lactation | 273.948 |
| Step 3 | phase of lactation × seropositivity to *B. besnoiti* | 276.812 |
| Step 4 | seropositivity to *B. besnoiti* | 274.837 |
| Step 5 | number of parturitions | 274.083 |

| **Table 4.19 –** Selection of final model by backward elimination according to the best Akaike's information criterion (AIC). The full model, having RDW (continuous variable) as response variable, contained the following independent variables: the clinical signs of bovine besnoitiosis (dichotomous variable: seronegative or asymptomatic seropositive animals vs. clinically affected cows), the number of parturitions (dichotomous variable: primiparous vs. multiparous cows), the phase of lactation (ordinal variable: early, mid, late, and dry), and all their two-way interactions. | | |
| --- | --- | --- |
| **Step of backward elimination** | **Eliminated variable** | **AIC** |
| Full model | -- | 285.853 |
| Step 1 | number of parturitions × clinical signs of bovine besnoitiosis | 283.853 |
| Step 2 | number of parturitions × phase of lactation | 280.213 |
| Step 3 | phase of lactation × clinical signs of bovine besnoitiosis | 282.986 |
| Step 4 | clinical signs of bovine besnoitiosis | 281.079 |
| Step 5 | number of parturitions | 274.083 |

| **Table 4.20 –** Selection of final model by backward elimination according to the best Akaike's information criterion (AIC). The full model, having Ca (continuous variable) as response variable, contained the following independent variables: the seropositivity to *B. besnoiti* (dichotomous variable: presence vs. absence of antibodies based on Western Blot results), the number of parturitions (dichotomous variable: primiparous vs. multiparous cows), the phase of lactation (ordinal variable: early, mid, late, and dry), and all their two-way interactions. | | |
| --- | --- | --- |
| **Step of backward elimination** | **Eliminated variable** | **AIC** |
| Full model | -- | 232.023 |
| Step 1 | number of parturitions × seropositivity to *B. besnoiti* | 230.110 |
| Step 2 | number of parturitions × phase of lactation | 226.653 |
| Step 3 | phase of lactation × seropositivity to *B. besnoiti* | 222.762 |
| Step 4 | seropositivity to *B. besnoiti* | 221.246 |
| Step 5 | phase of lactation | 217.610 |

| **Table 4.21 –** Selection of final model by backward elimination according to the best Akaike's information criterion (AIC). The full model, having Ca (continuous variable) as response variable, contained the following independent variables: the clinical signs of bovine besnoitiosis (dichotomous variable: seronegative or asymptomatic seropositive animals vs. clinically affected cows), the number of parturitions (dichotomous variable: primiparous vs. multiparous cows), the phase of lactation (ordinal variable: early, mid, late, and dry), and all their two-way interactions. | | |
| --- | --- | --- |
| **Step of backward elimination** | **Eliminated variable** | **AIC** |
| Full model | -- | 229.838 |
| Step 1 | number of parturitions × clinical signs of bovine besnoitiosis | 227.896 |
| Step 2 | number of parturitions × phase of lactation | 223.625 |
| Step 3 | phase of lactation × clinical signs of bovine besnoitiosis | 220.543 |
| Step 4 | clinical signs of bovine besnoitiosis | 218.971 |
| Step 5 | phase of lactation | 217.610 |

| **Table 4.22 –** Selection of final model by backward elimination according to the best Akaike's information criterion (AIC). The full model, having P (continuous variable) as response variable, contained the following independent variables: the seropositivity to *B. besnoiti* (dichotomous variable: presence vs. absence of antibodies based on Western Blot results), the number of parturitions (dichotomous variable: primiparous vs. multiparous cows), the phase of lactation (ordinal variable: early, mid, late, and dry), and all their two-way interactions. | | |
| --- | --- | --- |
| **Step of backward elimination** | **Eliminated variable** | **AIC** |
| Full model | -- | 340.007 |
| Step 1 | number of parturitions × seropositivity to *B. besnoiti* | 332.618 |
| Step 2 | phase of lactation × seropositivity to *B. besnoiti* | 330.159 |
| Step 3 | seropositivity to *B. besnoiti* | 328.828 |
| Step 4 | number of parturitions × phase of lactation | 316.033 |
| Step 5 | number of parturitions | 311.434 |

| **Table 4.23 –** Selection of final model by backward elimination according to the best Akaike's information criterion (AIC). The full model, having P (continuous variable) as response variable, contained the following independent variables: the clinical signs of bovine besnoitiosis (dichotomous variable: seronegative or asymptomatic seropositive animals vs. clinically affected cows), the number of parturitions (dichotomous variable: primiparous vs. multiparous cows), the phase of lactation (ordinal variable: early, mid, late, and dry), and all their two-way interactions. | | |
| --- | --- | --- |
| **Step of backward elimination** | **Eliminated variable** | **AIC** |
| Full model | -- | 337.096 |
| Step 1 | phase of lactation × clinical signs of bovine besnoitiosis | 330.843 |
| Step 2 | number of parturitions × clinical signs of bovine besnoitiosis | 329.347 |
| Step 3 | clinical signs of bovine besnoitiosis | 328.828 |
| Step 4 | number of parturitions × phase of lactation | 316.033 |
| Step 5 | number of parturitions | 311.434 |

| **Table 4.24 –** Selection of final model by backward elimination according to the best Akaike's information criterion (AIC). The full model, having NEFA (continuous variable) as response variable, contained the following independent variables: the seropositivity to *B. besnoiti* (dichotomous variable: presence vs. absence of antibodies based on Western Blot results), the number of parturitions (dichotomous variable: primiparous vs. multiparous cows), the phase of lactation (ordinal variable: early, mid, late, and dry), and all their two-way interactions. | | |
| --- | --- | --- |
| **Step of backward elimination** | **Eliminated variable** | **AIC** |
| Full model | -- | 39.105 |
| Step 1 | number of parturitions × seropositivity to *B. besnoiti* | 37.241 |
| Step 2 | phase of lactation × seropositivity to *B. besnoiti* | 33.417 |
| Step 3 | seropositivity to *B. besnoiti* | 31.612 |
| Step 4 | number of parturitions × phase of lactation | 33.303 |
| Step 5 | number of parturitions | 30.288 |

| **Table 4.25 –** Selection of final model by backward elimination according to the best Akaike's information criterion (AIC). The full model, having NEFA (continuous variable) as response variable, contained the following independent variables: the clinical signs of bovine besnoitiosis (dichotomous variable: seronegative or asymptomatic seropositive animals vs. clinically affected cows), the number of parturitions (dichotomous variable: primiparous vs. multiparous cows), the phase of lactation (ordinal variable: early, mid, late, and dry), and all their two-way interactions. | | |
| --- | --- | --- |
| **Step of backward elimination** | **Eliminated variable** | **AIC** |
| Full model | -- | 33.743 |
| Step 1 | phase of lactation × clinical signs of bovine besnoitiosis | 33.265 |
| Step 2 | number of parturitions × clinical signs of bovine besnoitiosis | 31.140 |
| Step 3 | seropositivity to *B. besnoiti* | 31.612 |
| Step 4 | number of parturitions × phase of lactation | 33.303 |
| Step 5 | number of parturitions | 30.288 |

| **Table 4.26 –** Selection of final model by backward elimination according to the best Akaike's information criterion (AIC). The full model, having AST (continuous variable) as response variable, contained the following independent variables: the seropositivity to *B. besnoiti* (dichotomous variable: presence vs. absence of antibodies based on Western Blot results), the number of parturitions (dichotomous variable: primiparous vs. multiparous cows), the phase of lactation (ordinal variable: early, mid, late, and dry), and all their two-way interactions. | | |
| --- | --- | --- |
| **Step of backward elimination** | **Eliminated variable** | **AIC** |
| Full model | -- | 77.848 |
| Step 1 | phase of lactation × seropositivity to *B. besnoiti* | 72.303 |
| Step 2 | number of parturitions × seropositivity to *B. besnoiti* | 70.815 |
| Step 3 | seropositivity to *B. besnoiti* | 38.728 |

| **Table 4.27 –** Selection of final model by backward elimination according to the best Akaike's information criterion (AIC). The full model, having AST (continuous variable) as response variable, contained the following independent variables: the clinical signs of bovine besnoitiosis (dichotomous variable: seronegative or asymptomatic seropositive animals vs. clinically affected cows), the number of parturitions (dichotomous variable: primiparous vs. multiparous cows), the phase of lactation (ordinal variable: early, mid, late, and dry), and all their two-way interactions. | | |
| --- | --- | --- |
| **Step of backward elimination** | **Eliminated variable** | **AIC** |
| Full model | -- | 69.107 |
| Step 1 | number of parturitions × clinical signs of bovine besnoitiosis | 67.124 |
| Step 2 | phase of lactation × clinical signs of bovine besnoitiosis | 63.664 |
| Step 3 | seropositivity to *B. besnoiti* | 38.728 |

| **Table 4.28 –** Selection of final model by backward elimination according to the best Akaike's information criterion (AIC). The full model, having CK (continuous variable) as response variable, contained the following independent variables: the seropositivity to *B. besnoiti* (dichotomous variable: presence vs. absence of antibodies based on Western Blot results), the number of parturitions (dichotomous variable: primiparous vs. multiparous cows), the phase of lactation (ordinal variable: early, mid, late, and dry), and all their two-way interactions. | | |
| --- | --- | --- |
| **Step of backward elimination** | **Eliminated variable** | **AIC** |
| Full model | -- | 768.209 |
| Step 1 | number of parturitions × seropositivity to *B. besnoiti* | 766.209 |
| Step 2 | phase of lactation × seropositivity to *B. besnoiti* | 760.353 |
| Step 3 | seropositivity to *B. besnoiti* | 740.220 |

| **Table 4.29 –** Selection of final model by backward elimination according to the best Akaike's information criterion (AIC). The full model, having CK (continuous variable) as response variable, contained the following independent variables: the clinical signs of bovine besnoitiosis (dichotomous variable: seronegative or asymptomatic seropositive animals vs. clinically affected cows), the number of parturitions (dichotomous variable: primiparous vs. multiparous cows), the phase of lactation (ordinal variable: early, mid, late, and dry), and all their two-way interactions. | | |
| --- | --- | --- |
| **Step of backward elimination** | **Eliminated variable** | **AIC** |
| Full model | -- | 782.591 |
| Step 1 | number of parturitions × clinical signs of bovine besnoitiosis | 780.591 |
| Step 2 | phase of lactation × clinical signs of bovine besnoitiosis | 776.620 |
| Step 3 | seropositivity to *B. besnoiti* | 740.220 |

| **Table 4.30 –** Selection of final model by backward elimination according to the best Akaike's information criterion (AIC). The full model, having LDH (continuous variable) as response variable, contained the following independent variables: the seropositivity to *B. besnoiti* (dichotomous variable: presence vs. absence of antibodies based on Western Blot results), the number of parturitions (dichotomous variable: primiparous vs. multiparous cows), the phase of lactation (ordinal variable: early, mid, late, and dry), and all their two-way interactions. | | |
| --- | --- | --- |
| **Step of backward elimination** | **Eliminated variable** | **AIC** |
| Full model | -- | 525.509 |
| Step 1 | number of parturitions × seropositivity to *B. besnoiti* | 523.678 |
| Step 2 | phase of lactation × seropositivity to *B. besnoiti* | 518.465 |
| Step 3 | seropositivity to *B. besnoiti* | 516.495 |
| Step 4 | number of parturitions × phase of lactation | 511.905 |
| Step 5 | phase of lactation | 480.299 |

| **Table 4.31 –** Selection of final model by backward elimination according to the best Akaike's information criterion (AIC). The full model, having LDH (continuous variable) as response variable, contained the following independent variables: the clinical signs of bovine besnoitiosis (dichotomous variable: seronegative or asymptomatic seropositive animals vs. clinically affected cows), the number of parturitions (dichotomous variable: primiparous vs. multiparous cows), the phase of lactation (ordinal variable: early, mid, late, and dry), and all their two-way interactions. | | |
| --- | --- | --- |
| **Step of backward elimination** | **Eliminated variable** | **AIC** |
| Full model | -- | 564.158 |
| Step 1 | number of parturitions × clinical signs of bovine besnoitiosis | 562.227 |
| Step 2 | phase of lactation × clinical signs of bovine besnoitiosis | 559.612 |
| Step 3 | seropositivity to *B. besnoiti* | 516.495 |
| Step 4 | number of parturitions × phase of lactation | 511.905 |
| Step 5 | phase of lactation | 480.299 |
